# Inhibitor Trapping in N-Myristoyltransferases as a Mechanism for Drug Potency

**DOI:** 10.3390/ijms241411610

**Published:** 2023-07-18

**Authors:** Danislav S. Spassov, Mariyana Atanasova, Irini Doytchinova

**Affiliations:** Department of Chemistry, Faculty of Pharmacy, Medical University of Sofia, 1000 Sofia, Bulgaria; matanasova@pharmfac.mu-sofia.bg (M.A.); idoytchinova@pharmfac.mu-sofia.bg (I.D.)

**Keywords:** NMT, N-myristoyltransferases, conformational stability and dynamics, predicting inhibitor activity, drug design, mechanism of inhibition, inhibitor trap, inhibitor potency, enzyme inhibition, drug potency

## Abstract

Predicting inhibitor potency is critical in drug design and development, yet it has remained one of computational biology’s biggest unresolved challenges. Here, we show that in the case of the N-myristoyltransferase (NMT), this problem could be traced to the mechanisms by which the NMT enzyme is inhibited. NMT adopts open or closed conformations necessary for orchestrating the different steps of the catalytic process. The results indicate that the potency of the NMT inhibitors is determined by their ability to stabilize the enzyme conformation in the closed state, and that in this state, the small molecules themselves are trapped and locked inside the structure of the enzyme, creating a significant barrier for their dissociation. By using molecular dynamics simulations, we demonstrate that the conformational stabilization of the protein molecule in its closed form is highly correlated with the ligands activity and can be used to predict their potency. Hence, predicting inhibitor potency in silico might depend on modeling the conformational changes of the protein molecule upon binding of the ligand rather than estimating the changes in free binding energy that arise from their interaction.

## 1. Introduction

Predicting the binding affinity of small molecule ligands to proteins is of particular importance in drug development, and in recent years docking has gained widespread use in drug design as a computation method to identify small molecules with desirable activity [1,2,3]. Molecular docking has two main goals, namely predicting the pose of a ligand in its binding site and calculating the change in free binding energy expressed as a docking score for each pose [1,2,3]. So far, the existing docking tools are generally able to generate the correct binding pose; however, docking scores have been found to be uncorrelated with empirically determined binding affinity and inhibitor potency [4,5,6,7]. The inaccuracy of the scoring functions has been attributed to approximation introduced in the docking algorithms in calculations of some parameters required for the determination of the change in free binding energy (e.g., entropy, solvation, flexibility, or polarization), leading to misestimation of the strength of certain bonds [5,8,9], however, in fact, the precise reasons for this have remained not well understood. The unreliability of docking to identify compounds with high potency creates a dilemma that is difficult to resolve at the moment and represents a significant obstacle to drug development.

**Figure 1:**
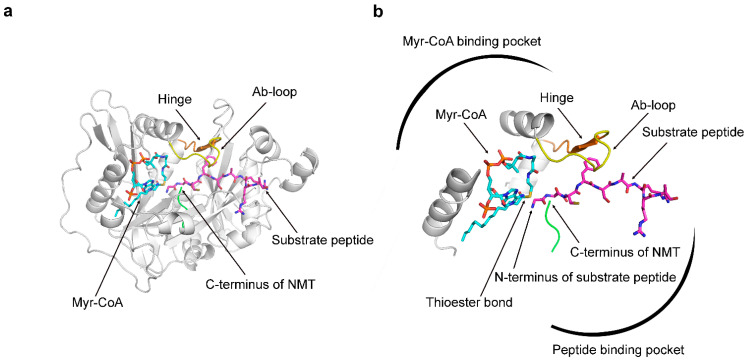
The 3D structure of NMT. The images depict the ternary HsNMT1: Myr-CoA: Substrate peptide complex (PDB 6QRM, monomer B). (**a**) A view of the whole molecule; (**b**) A zoomed view of the catalytic centre. NMT contains two adjacent binding pockets for binding the cofactor Myr-CoA, shown in cyan, and for the substrate peptide, shown in magenta. In this binding mode, the N-terminus of the substrate peptide is near the thioester bond of Myr-CoA, facilitating the transfer of myristic acid from Myr-CoA to the peptide during the myristoylation reaction. Access to the peptide binding pocket is controlled by the Ab-loop shown in yellow. The hinge region, described for the first time in this study, is shown in orange and regulates the opening and closing of the Ab-loop. The C-terminus of NMT, shown in green, is located in the enzyme’s active site, and its free carboxyl group serves as a catalytic base during the reaction.

We began this study by attempting to understand why docking scores are not correlated with the activity of the N-myristoyltransferases (NMT) inhibitors. NMTs belong to an evolutionarily ancient enzyme family that is present in all metazoan species and is represented in humans by two members—NMT1 and NMT2 that catalyze the transfer of myristic acid (a 14-carbon atom fatty acid) to the N-termini of specific cellular proteins [10,11,12]. Myristoylation facilitates the attachment of the modified proteins to the cellular membranes and performs key roles in regulating cell signaling, protein trafficking, and localization [12,13,14]. The catalytic center of NMT contains two binding pockets (Figure 1). The peptide binding pocket is the binding site for the substrate peptide, which in the cellular context is the peptide sequence at the N-terminal region of the myristoylated proteins [14,15,16,17]. The other pocket, located proximally to the first, is the binding site for the cofactor Myristoyl-Coenzyme A (Myr-CoA) [14,15,16,17]. This binding mode allows the transfer of myristic acid from Myr-CoA to the N-terminus of the substrate protein, while CoA is released as a byproduct of the reaction [14].

The myristoylation reaction is a multistep process that is initiated by a nucleophilic attack of the deprotonated N-terminus of the substrate peptide on the thioester bond of Myr-CoA, and the whole process is orchestrated through complex conformational changes in the enzyme that regulate the different steps, such as binding of the substrates, formation of an intermediary complex, and dissociation of the products of the reactions [15]. A series of crystal structures of complexes of NMT with its substrates, intermediate complexes, or products has revealed significant insight into the mechanism of catalysis [15]. The Ab-loop of NMT, positioned just above the peptide binding pocket (Figure 1), plays a key regulatory role in this process [15]. The Ab-loop can exist in two conformational states—closed and open and functions as a lid that controls the accessibility of the active site [15]. The closing of the Ab-loop leads to the formation of the ceiling of the active site and is required for the generation of the catalytically active conformation [15]. On the other hand, the opening of the Ab-loop is necessary for the initial binding of the substrate peptide and the consequent dissociation of the product of the reaction—the myristoylated peptide [15].

**Figure 2:**
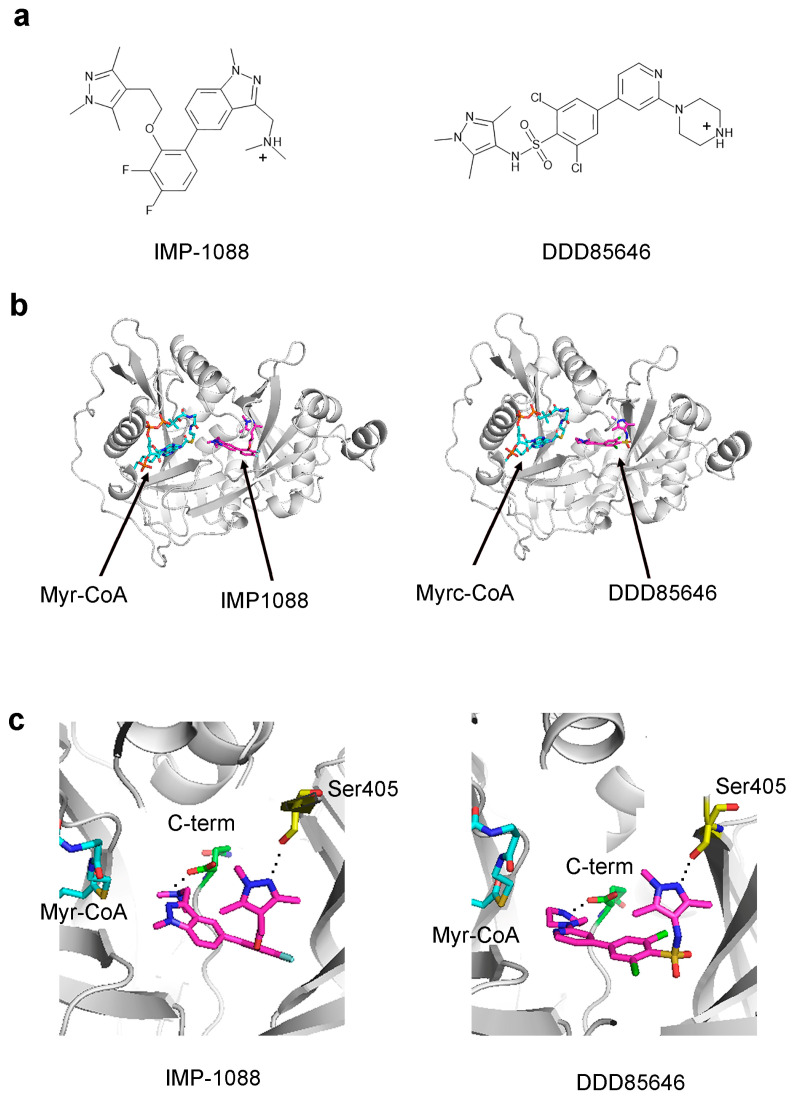
Structures and binding modes of the NMT inhibitors IMP-1088 and DDD85646. (**a**) The 2D structures of IMP-1088 and DDD85646 are shown. The compounds are positively charged at neutral pH, as indicated: (**b**) IMP-1088 (PDB 5MU6) and DDD85646 (PDB 3IWE) interact within the peptide binding pocket of NMT; (**c**) IMP-1088 and DDD85646 form a salt bridge through their positively charged chemical groups with the negatively charged C-terminus of NMT and a hydrogen bond with Ser405 through their pyrazole rings.

NMT inhibitors that bind into the peptide binding pocket of NMT have been developed. The most prominent of them—the pyrazole containing DDD85646 and IMP-1088—have been reported to display potent activity in the nanomolar and the picomolar range, respectively [16,17]. Although their structures differ substantially, the two inhibitors display a common interaction mode, including the formation of a salt bridge between the positively charged piperazine ring in DDD85646 or the dimethylamino group in IMP-1088 and the negatively charged C-terminus of NMT, as well as a hydrogen bond with Ser405 (Figure 2) [18,19].

The NMT inhibitors are promising compounds that may find application in treating parasitic infections such as sleeping sickness, malaria, and leishmaniasis [20,21,22]. In addition, by inhibiting myristoylation of important proto-oncogenes like c-Src, c-Abl, or others, the NMT inhibitors display potent anti-tumor activity and have produced complete anti-tumor responses in preclinical murine models [23,24,25,26,27].

## 2. Results

### 2.1. Docking and Inhibitor Potency

Recently, we performed a virtual screening to identify novel NMT inhibitors [19]. By screening over 1.1 million structures downloaded from the ZINC database [28], 24 compounds were selected based on their docking scores in AutoDock Vina v.1.1.2 (The Scripps Research Institute) [29] and GOLD v.5.3.0 (The Cambridge Crystallographic Data Centre, CCDC) [30], and their potency was determined by a fluorogenic NMT assay (Table S1) [31]. Seven of these compounds displayed micromolar activity with IC_50_ between 14 and 97 µM, while the remaining seventeen had IC_50_ > 100 µM (Table 1 and Table S1). Although the best hits from the virtual screening received higher or at least comparable docking scores with the potent NMT inhibitors DDD85646 and IMP-1088, they were found to be thousands of times less active (Table 1).

Since docking scores are known to be unreliable in the identification of compounds with high potency [4,5,6,7], the NMT complexes were further analyzed by molecular dynamics (MD) simulations, followed by calculations of the change in free binding energy resulting from the bonding between the ligand and the protein using the MM-PBSA (Molecular Mechanic/Poisson–Boltzmann surface area) method [32]. However, performing these calculations on the NMT-ligand complexes reveals no correlation between the change in MM-PBSA energy and inhibitory potency (Table 1, last column). For example, it was observed that some ligands that are over thirteen thousand-fold less active than the control NMT inhibitors displayed more considerable negative changes in MM-PBSA binding energies (Table 1, compare compounds **10** and **11** with IMP-1088). According to the model of competitive inhibition, the ligands compete for binding to the enzyme’s active site, and their binding affinity is determined by the change in free binding energy [5]. X-ray structures demonstrate that the NMT inhibitors DDD85646 and IMP-1088 occupy the peptide binding pocket of NMT and compete with the substrate peptide for binding. (Figure S1), which is also confirmed by kinetic studies for DDD85646 [16]. The crystallographic structures show that the substrate peptide forms more numerous interactions within the enzyme’s binding site than the highly potent NMT inhibitors DDD85646 and IMP-1088 (Figure S2). Consistent with this, the substrate peptide receives a higher docking score in GOLD, a more negative AutoDock Vina (ADV) affinity score, and a more negative change in free binding energy by MM-PBSA than the potent NMT inhibitors (Table 1, compare row 12 with row 1), suggesting that the substrate peptide interacts more strongly with NMT than the potent NMT inhibitors and hence should be able to outcompete them. This paradoxical prediction is unlikely to be related to incorrect calculations performed by the docking algorithms because it can be traced to the experimentally determined X-rays structures (Figure S2), implying that it is due to a biological/chemical mechanism. Hence, this may suggest the hypothesis that the inhibitor potency is not determined solely by the change in free binding energy, as assigned during calculations of the docking scores.

### 2.2. The Synergy between Complementary Fragments

**Figure 3:**
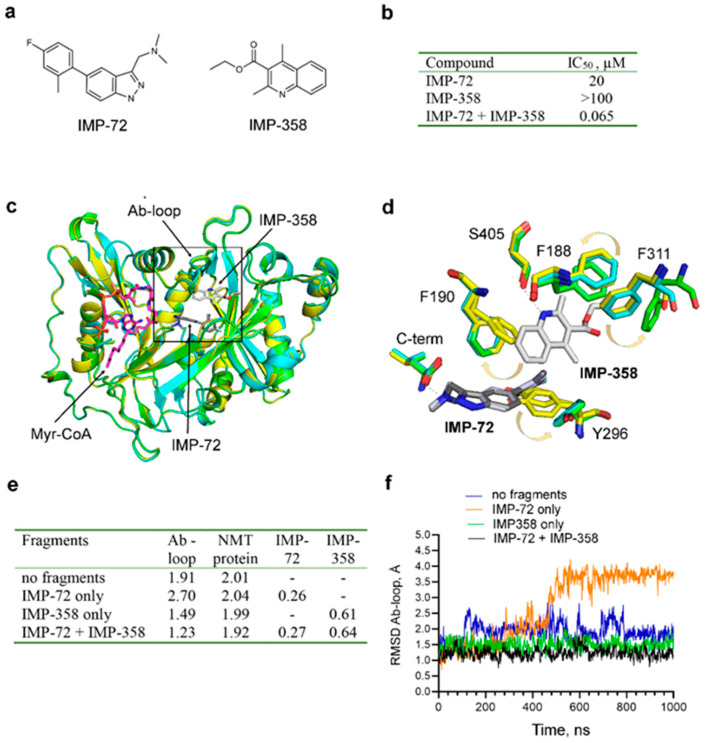
Fragment synergy could be due to the stabilization of the Ab-loop in its closed state; (**a**) Structures of IMP-72 and IMP-358, intermediate fragments in the development of IMP-1088; (**b**) IC_50_ of IMP-72 or IMP-358 as single agents or IMP-72 in the presence of 100 µM IMP-358. The fragments display remarkable synergism; (**c**) Structural superimposition of binary PvNMT: Myr-CoA (PDB 4B10, yellow), ternary PvNMT: Myr-CoA: IMP-72 (PDB 5O48, cyan), and quaternary PvNMT: Myr-CoA: IMP-72: IMP-358 (PDB 5O4V, green) complexes. The carbon atoms of IMP-358 are shown in light grey, and those of IMP-72 in a darker shade of grey; (**d**) A zoomed view of the boxed region in the image on the left. Arrows indicate conformational movements of the depicted residues due to the binding of the fragments. The figure shows that all major conformational changes are proximal and that the binding of IMP-72 does not induce distal conformational changes in the binding pocket of IMP-358 and vice versa. The numbering corresponds to the position of the amino acid residues in HsNMT1. Y296 corresponds to Y211, F188 to F103, F190 to F105, F311 to F226, and S405 to S319 in PvNMT; (**e**) Average RMSD in angstroms (Å) of the heavy atoms of the Ab-loop, the whole NMT protein, IMP-72, and IMP-358 based on MD simulations of the crystal structure PDB 5O4V, either in the absence of fragments or in the presence of IMP-72, IMP-358, or both of them; (**f**) RMSD values of the heavy atoms of the Ab-loop in the time course of MD simulations. RMSD < 1.5 Å indicates that the Ab-loop remains locked in its closed conformation by IMP-358.

During the development of IMP-1088, two intermediary fragments were identified that occupy non-overlapping regions in the peptide binding pocket of NMT (Figure 3) [17].

One of the fragments, IMP-72, binds to the C-terminal carboxylate of NMT and has an IC_50_ of 20 µM. The other fragment, IMP-358, forms a hydrogen bond with Ser405 at a complementary region and has an IC_50_ >100 µM. Remarkably, performing NMT assays in the presence of 100 µM IMP-358 reduces the IC_50_ of IMP-72 from 20 µM to 65 nM, or 308 fold (Figure 3b) [17]. The synergism between complementary fragments is not unique to IMP-1088; but it was also observed during the development of the DDD85646 series of compounds [16]. Although convincingly demonstrated experimentally, this synergism cannot be easily explained by the current theory of enzyme inhibition. For example, docking scores are calculated by summing up the individual contributions of the interactions between the ligand and the protein. Thus, complementary fragments are expected to have additive and not synergistic effects, and only provided that the fragments are covalently attached to each other. The fact that synergism is observed even when the two fragments are not covalently connected may also appear difficult to explain, as no theoretical basis for this observation can be provided.

Indeed, the mechanisms that mediate this synergy are not considered in the docking programs’ algorithms. This was confirmed by using an in silico experiment, where the structure of IMP-1088 was split into two fragments: fragment C, which binds to the C-terminus of NMT, and fragment S, which binds to Ser405 (Figure S3). It was found that the docking scores of IMP-1088 was approximately the sum of the docking score of the individual fragments (Figure S3). Therefore, the failure of docking to correctly predict the potency of NMT inhibitors may be due to the fact that it does not account for the observed synergism.

Elucidating the mechanism that mediates synergy may give important insights into the mechanism of enzyme inhibition and how to model it in silico. One possibility is that the binding of one fragment induces conformational changes in the binding site of the other fragment to increase its affinity. To address this question, we superimposed the NMT crystallographic structures in the absence of fragments (PDB 4B10), in complex with IMP-72 alone (PDB 5O48), or in complex with both IMP-72 and IMP-358 (PDB 5O4V), which had been experimentally determined previously [17,33]. However, all conformational changes were found to be attributable to the direct binding of the fragments and not to occur distantly (Figure 3c,d). For instance, the binding of IMP-72 pushes Y296 towards a new position but does not affect residues that interact with IMP-358 (Figure 3d). Similarly, the binding of IMP-358 affects the positions of residues in its nearby environment, such as Phe311, Phe188, and Phe190, but has no effect on residues interacting directly with IMP-72 (Figure 3d).

An alternative hypothesis is that a fragment may stabilize a specific pre-existing enzyme conformation or a dynamic state, which in turn may indirectly augment the potency of the other fragment. Providing evidence for this hypothesis required the analysis of different NMT conformational states. In this aspect, alternative conformations have been reported in the literature in relation to the mechanism of NMT catalysis and have suggested an essential role for the opening and closing of the Ab-loop of NMT in regulating the accessibility of the catalytic site [15]. To understand the mechanism of the observed synergy, we performed MD simulations using the crystallographic structure of the NMT complex with the two fragments IMP-72 and IMP-358 (PDB 5O4V). The MD simulations were performed either in the absence of the fragments, in the presence of only IMP-72, in the presence of only IMP-358, or in the presence of both fragments. RMSD trajectory values were determined for the Ab-loop, the whole NMT protein, and the fragments (Figure 3e,f). The results indicate that the binding of IMP-358 leads to immobilization and tethering of the Ab-loop in its closed conformation (RMSD < 1.5 Å) (Figure 3e,f). In contrast, in the absence of IMP-358, the Ab-loop is mobile (RMSD > 1.5 Å), and in the presence of IMP-72, it adopts an open conformation (RMSD = 2.70) (Figure 3e,f). IMP-358 interacts with Ser405 and aromatic residues (Phe190, Phe188, and Phe311) at the base of the Ab-loop, potentially explaining the observed results (Figure 3d). Since the opening of the Ab-loop is required for dissociation of the products of the NMT reaction [15], its stabilization in closed conformation may also prevent the dissociation of the bound fragments and hence may provide an explanation for the observed synergistic effect.

### 2.3. The NMT Inhibitors Are Bound and Entrapped Inside the Closed Enzyme Conformation

**Figure 4:**
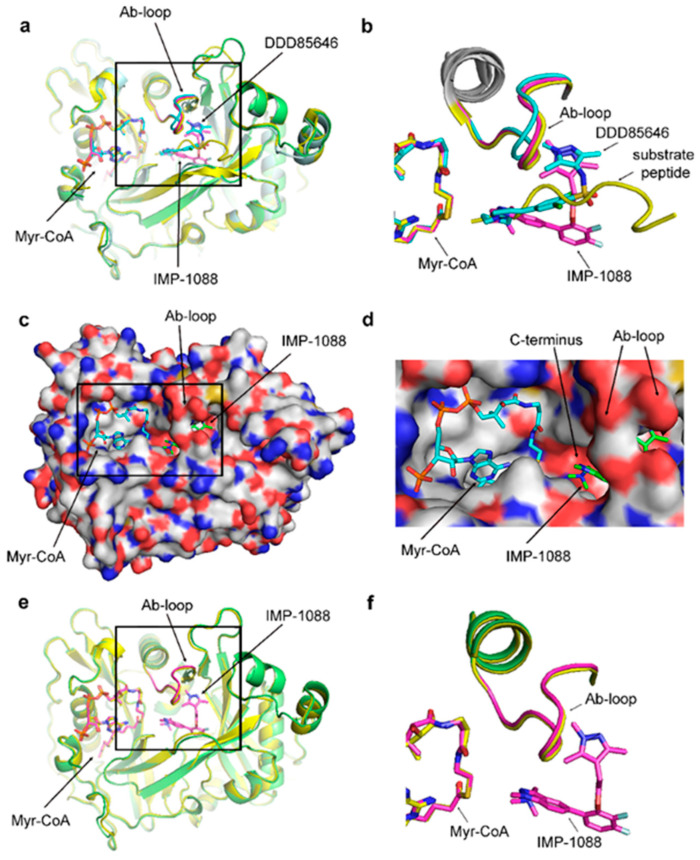
NMT inhibitors are bound inside the closed NMT conformation. The whole NMT protein structures are displayed on the panels on the left, and zoomed views of the boxed areas are shown on the right. (**a**,**b**) Superimposition of the crystal structures of HsNMT1 in complex with a substrate peptide (PDB 6QRM, yellow) or the NMT inhibitors DDD85646 (PDB 3IWE, cyan) and IMP-1088 (PDB 5MU6, green; IMP-1008 and Ab-loop highlighted in magenta) is shown. In all structures, the Ab-loop is in a closed conformation; (**c**,**d**) the surface representation of the ternary complex of HsNMT1: IMP-1088: Myr-CoA, based on crystal structure PDB 5MU6, is depicted. IMP-1088 (green) is partially visible and bound inside the closed conformation of NMT; (**e**,**f**) Superimposition of the crystal structures of HsNMT1: Myr-CoA binary complex (PDB 3IU1, yellow) and HsNMT1: Myr-CoA: IMP-1088 ternary complex (PDB 5MU6, green; IMP-1008, Myr-CoA, and Ab-loop are highlighted in magenta).

The role of the opening and closing of the Ab-loop in orchestrating the different stages of the enzyme reaction is well described [15]. However, the NMT inhibitors’ interaction with these different enzyme conformations is much less understood. Superimposition of the crystal structure of NMT in complex with substrate peptide (PDB 6QRM), which has been previously reported to adopt a closed conformation [15], with the crystallographic structures of complexes of NMT with DDD85646 (PDB 3IWE) and IMP-1088 (PDB 5MU6) reveals that both NMT inhibitors are bound to the closed enzyme conformation (Figure 4a,b).

A surface representation of the NMT complex with IMP-1088 (PDB 5MU6) reveals that the inhibitor is buried and bound inside the structure of the enzyme, with the Ab-loop falling on top of the small molecule (Figure 4c,d). The closed NMT conformation is not induced or created by the presence of the inhibitors because superimposition with the crystallographic structure of the NMT in the absence of NMT inhibitors (PDB 3IU1) reveals a very similar closed enzyme conformation (Figure 4e,f). This suggests that the opening of the Ab-loop may be required to accommodate the NMT inhibitors in the binding site, followed by its closure on top.

### 2.4. The NMT Inhibitors Interact with the Hinge Region of the Ab-Loop

**Figure 5:**
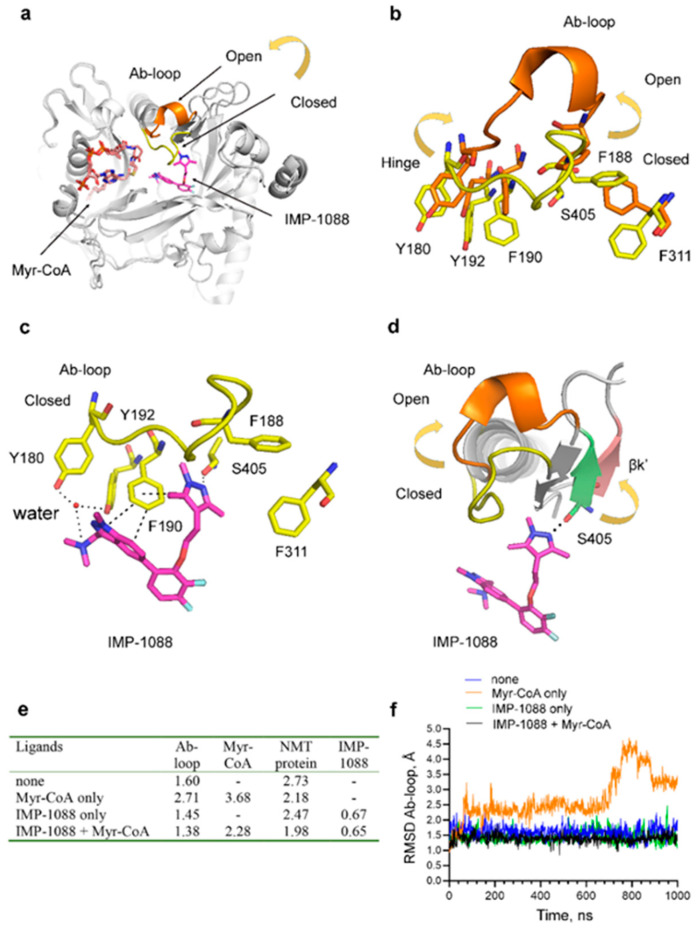
NMT inhibitors tether the Ab-loop in the closed conformation. (**a**–**d**) Structural superimposition of binary ScNMT: Myr-CoA complex (PDB 1IIC) with Ab-loop in the open configuration (orange) and ternary HsNMT1: Myr-CoA: IMP-1088 complex (PDB 5MU6) with Ab-loop in the closed conformation (yellow); (**a**) Whole NMT molecule; (**b**) A zoomed view of the open and closed Ab-loop conformations. The opening of the Ab-loop is accompanied by significant changes in the positions of the depicted aromatic phenylalanine and tyrosine residues at the base of the loop. Residues from the open NMT conformation are shown in orange and from the closed in yellow; (**c**) Interactions between IMP-1088 and residues at the hinge region of the closed Ab-loop include stacking interactions with F190 and a water bridge with Y192. The inhibitor also forms a hydrogen bond with Ser405 and a water bridge with Y180 from the Ab-loop. In the open conformation of the Ab-loop, these residues are pushed up and not available for interaction with the inhibitor; (**d**) The opening of the Ab-loop is accompanied by rotation of the βk′ sheet, where S405 is located. The position of the βk′ sheet in the structure with an open Ab-loop is shown in strawberry and in the structure with a closed Ab-loop in green. The formation of a hydrogen bond between the inhibitor and S405 is expected to hinder this movement and stabilize the closed Ab-loop conformation; (**e**) Average RMSD in angstroms (Å) of the heavy atoms of the Ab-loop, Myr-CoA, the entire NMT protein, and IMP-1088, based on MD simulations of the Apo NMT enzyme (none) or its binary or ternary complexes with Myr-CoA and IMP-1088; (**f**) RMSD values of the heavy atoms of the Ab-loop in the time course of MD simulations. RMSD < 1.5 Å indicates that the Ab-loop remains locked in its closed conformation, and RMSD > 1.5 Å indicates that it opens.

Superimposition of crystal structures of NMT in open (PDB 1IIC) or closed (PDB 5MU6) conformation demonstrates that opening of the Ab-loop is accompanied by a significant shift in the position of several phenylalanine and tyrosine residues, including Tyr180, Phe188, Phe190, Tyr192, and Phe311 (Figure 5a,b).

Tyr180 is the first amino acid of the Ab-loop, while Phe188, Phe190, and Tyr192 are located in the small βb-sheet and bB-loop at the Ab-loop’s base (Figure S4). These aromatic residues form part of the ceiling of the peptide binding pocket of NMT. The closure of the Ab-loop brings these residues to the active site, and its opening moves them away (Figure 5b and Figure S5b). Considering the role of hydrophobic aromatic residues in protein folding, these residues likely function as a hinge that controls the opening and closing of the Ab-loop (Figure S4). The NMT inhibitors IMP-1088 and DDD85646 interact directly with residues from this hinge region, such as Phe190, with which they form triple and double stacking interactions, respectively, and Y192, which is bound to the inhibitors through a structural water molecule (Figure 5c and Figure S5d). As previously described, the same residues are implicated in mediating synergy between the fragments IMP-358 and IMP-72 (Figure 3d). The NMT inhibitors do not interact with residues from the Ab-loop, except for Y180, to which they are bound through a structural water molecule (Figure 5c and Figure S5d). Both DDD85646 and IMP-1088 form hydrogen bonds with Ser405 [19], raising questions about the significance of this residue. Ser405 is located at the base of a small βk′ sheet proximal to the Ab-loop. Superimposition of NMT crystal structures with open or closed Ab-loops reveals that the opening of the Ab-loop is accompanied by translational rotation of the βk′ sheet, which moves away from the binding site (Figure 5d and Figure S5c). Hence, the formation of a hydrogen bond between the inhibitor and Ser405 may hinder this movement and stabilize the closed Ab-loop conformation. All of these imply that binding the inhibitors may prevent the opening of the Ab-loop and stabilize its closed conformation. A particular role in this may have the inhibitors’ pyrazole ring, which participates in a hydrogen bond with Ser405 and π-π stacking interactions with Phe190 from the hinge region. This dual interaction may help tether the Ab-loop in its closed position. In addition, we studied the interaction between the inhibitors and NMT through docking. Docking of DDD85646 in the closed NMT conformation reproduces the pose and the interactions in the binding site observed in the crystallographic structures, including the salt bridge with the protein C-terminus, hydrogen bond with Ser405, and stacking interactions with Phe190 (Figure S6). When DDD85646 is docked into the open conformation, the small molecule adopts a more extended conformation. The salt bridge with the C-terminus is formed, but the Ser405 and Phe190 interactions with the pyrazole ring are not preserved (Figure S6). Notably, the docking score of DDD85646 in the open conformation is dramatically lower compared to the closed conformation (ChemPLP score 64.41 vs. 103.59) (Figure S6), implying that the interaction with the open form is not optimal and that a more energetically favorable interaction will be within the closed conformation of the enzyme.

### 2.5. The Potent NMT Inhibitors Stabilize the Closed Enzyme Conformation

The observation that the potent NMT inhibitors IMP-1088 and DDD85646 interact with residues involved in the opening or closing of the Ab-loop suggests that these ligands may lock the position of this loop in the closed conformation. To address this question, we performed MD simulations using the NMT structure in the absence of Myr-CoA and inhibitors, its binary complex with Myr-CoA, the binary complex with IMP-1088, and the ternary complex with both Myr-CoA and IMP-1088 (Figure 5e,f). In the absence of ligands, the Ab-loop is slightly mobile (RMSD = 1.60), but the binding of Myr-CoA leads to the opening of the Ab-loop (RMSD = 2.71) (Figure 5e,f), possibly as a mechanism that allows the consequent binding of the substrate peptide during the enzyme reaction. However, the binding of IMP-1088 locks the Ab-loop in its closed conformation (RMSD < 1.5 Å), both in the presence or absence of the cofactor Myr-CoA (Figure 5e,f). Similarly, DDD85646 locks the Ab-loop in its closed position (RMSD < 1.5 Å) during MD simulations of its ternary complex with NMT and Myr-CoA. The results also indicate the existence of crosstalk between the NMT inhibitors, the cofactor Myr-CoA, and the NMT protein. For example, it was found that the presence of IMP-1088 stabilizes the complex between NMT and Myr-CoA (Figure S7a,b) and that the presence of Myr-CoA exerts some positive effect on the stability of the IMP-1088-NMT complex (Figure S7c,d), consistent with experimental observations that demonstrate cooperativity of binding between NMT inhibitors and the cofactor Myr-CoA [16]. Considering that there is no direct interaction between IMP-1088 and Myr-CoA, the observed effects could be attributed to the stabilization of the conformation of the NMT protein by the presence of the small molecules. In agreement with this, both Myr-CoA and IMP-1088 were found to reduce the conformational mobility of the NMT protein during MD simulations (Figure S7e,f).

### 2.6. The Modest NMT Inhibitors Destabilize the Closed Enzyme Conformation

For the NMT inhibitors displaying high potency, we find that (a) they lock the Ab-loop in its closed conformation, (b) stabilize the complex between NMT and Myr-CoA, and (c) constrain the mobility of the NMT protein as a whole (Figure 5). Here, we asked the question of whether we can observe analogous conformational stabilization during MD simulations for ligands that are not optimized for inhibitory activity experimentally, such as the ones that are obtained by virtual screening. To address this question, we performed MD simulations on NMT complexes of the 24 ligands identified by virtual screening (Table S1). As described previously, these ligands have comparable or even higher docking scores but display significantly reduced potency compared to the control NMT inhibitors (Table 1 and Table S1).

**Figure 6:**
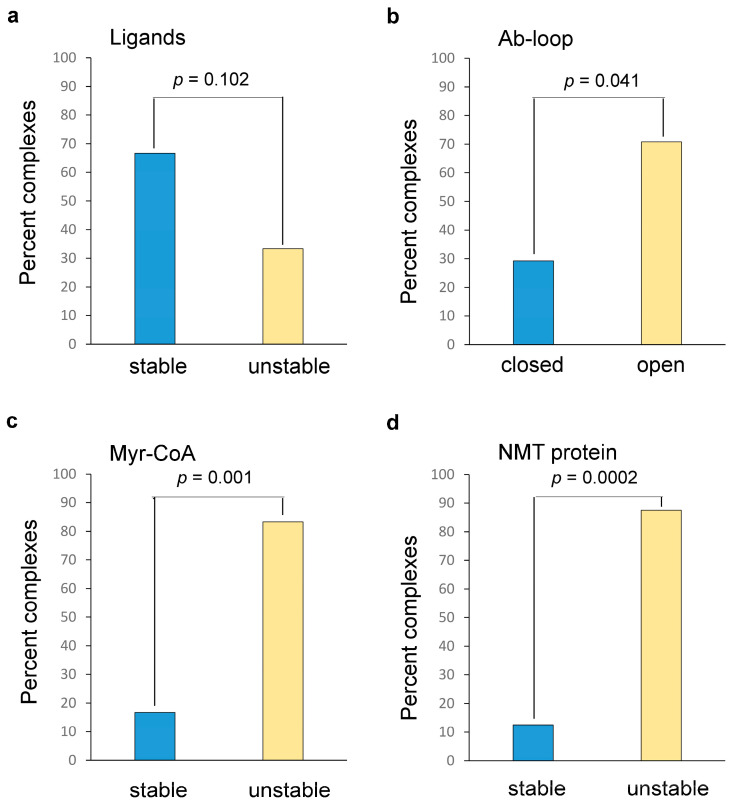
The Ab-loop, My-CoA, and NMT protein dynamics are significantly related to the ligands’ activity. The graphs depict the distribution of RMSD of the indicated parameters in the NMT complexes with the 24 ligands identified by virtual screening, relative to the complexes of the control NMT inhibitors; (**a**) Percent of stable or unstable NMT-ligand complexes based on the average RMSD of the ligands. RMSD of the ligands < 1.5 Å indicates a stable complex; RMSD > 1.5 Å indicates an unstable complex. There is no statistically significant relationship between the activity of the ligands and whether their RMSD is below or above 1.5 Å (Chi-square test, *p* > 0.05); (**b**) Percent complexes in which the Ab-loop remains closed or opens. The Ab-loop is considered to be in its closed conformation if its average RMSD is equal to or lower than the average RMSD of the Ab-loop in the control inhibitors’ complexes (RMSD < 1.5 Å) and in its open conformation if its mobility is increased above these values (RMSD > 1.5 Å). The conformational mobility of the Ab-loop is significantly related to the ligand potency (Chi-square test, *p* < 0.05); (**c**) Percent complexes with either an equal or decreased (stable) or increased (unstable) average RMSD of Myr-CoA relative to the NMT complexes of IMP-1088 and DDD85646. The stability of the Myr-CoA complex with NMT is significantly related to the potency of the ligands (Chi-square test, *p* = 0.001); (**d**) Percent complexes with equal or decreased (stable) or increased (unstable) average RMSD of NMT protein relative to the complexes of the control NMT inhibitors. The dynamics of the NMT protein are significantly related to the potency of the ligands (Chi-square test, *p* < 0.001).

The RMSD of the ligands during MD simulations may provide information about the stability of the protein-ligand complexes and the compounds’ activity. Typically, a stable complex in MD simulations is thought to occur when the RMSD of the ligand is below 1.5 Å. Consistent with this, the RMSD of the potent control NMT inhibitors was found to be less than 1.5 Å. Among the NMT complexes with the ligands from the virtual screening, one-third display instability in the position of the ligand (RMSD > 1.5 Å) (Figure 6a), potentially explaining their weak activity.

However, the remaining two-thirds of the compounds form stable complexes with NMT during MD simulations (RMSD < 1.5 Å) (Figure 6a), similar to IMP-1088 and DDD85646, indicating that this approach does not significantly differentiate between the modest and the potent NMT inhibitors (Chi-square test, *p* > 0.05) (Figure 6a). Thus, using the RMSD of the ligands as the only parameter in the analysis is not sufficient to discern the highly potent inhibitors from those that have weak activity.

**Figure 7:**
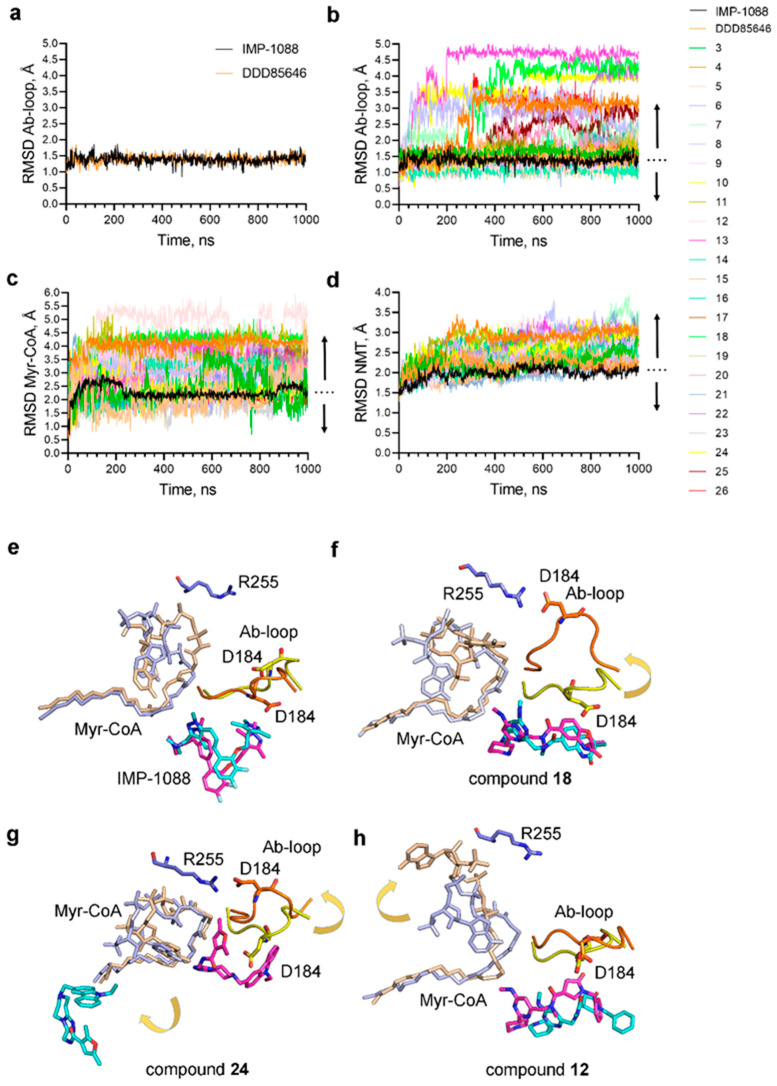
Conformational changes induced by binding of ligands to HsNMT1 based on MD simulations; (**a**–**d**) RMSD of the heavy atoms of the Ab-loop, Myr-CoA, or NMT protein during the time course of MD simulations for the NMT complexes with IMP-1088, DDD85646, and the 24 ligands (compound **3** to compound **26**, the legend on the right). RMSD values in the IMP-1088 complexes are indicated in black. The up arrow indicates complexes with increased dynamics (RMSD) compared to the NMT complex of IMP-1088; the down arrow shows complexes with decreased mobility of the indicated Y-axis parameters; (**a**) The potent NMT inhibitors IMP-1088 and DDD85646 lock the Ab-loop in a closed conformation during the entire duration of MD simulations, RMSD < 1.5 Å; (**b**) RMSD of the Ab-loop; (**c**) RMSD of Myr-CoA; (**d**) RMSD of NMT protein; (**e**–**h**) Ab-loop opening and displacement of Myr-CoA during MD simulation of selected NMT-ligand complexes. Structural superimposition of the frames at the beginning (0 ns) and the end (1000 ns) of the MD simulations is shown. In time 0, the ligand is shown in magenta, the Ab-loop in yellow, and Myr-CoA in light blue; in time point 1000 ns, the ligand is shown in cyan, the Ab-loop in orange, Myr-CoA in wheat, and R255 is in blue; (**e**) In the HsNMT1: IMP-1088 complex, the Ab-loop remains closed, and the cofactor is confined to its binding pocket; (**f**) In the HsNMT1: compound **18** complex, the Ab-loop opens, leading to the formation of a salt bridge between R255 and D184; (**g**) In the HsNMT1: compound **24** complex, the Ab-loop opens, and the ligand is completely displaced from the binding site; (**h**) In the HsNMT1: compound **12** complex, the Ab-loop remains closed, but Myr-CoA is partially displaced from its binding pocket.

That is why we searched for other parameters (RMSD values) obtained during the MD simulations that may correlate better with the activity of the ligands. Considering the Ab-loop’s suggested role in the ligands’ entrapment, we investigated how the different ligands affect its mobility. As expected, IMP-1088 and DDD85646 immobilize the Ab-loop of NMT in its closed conformation (RMSD < 1.5 Å) (Figure 7a, Movie S1).

In contrast, 70.8% of the NMT complexes with ligands from the virtual screening show increased RMSD of the Ab-loop compared to the control inhibitors (RMSD > 1.5 Å), indicating that it displays increased conformational mobility (Figure 6b and Figure 7b). In many cases, the binding of the small molecule leads to the complete opening of the Ab-loop and the consequent formation of a salt bridge between Asp184, located in the middle of the Ab-loop, and Arg255 (Figure 7f,g, Movies S2 and S3). The conformational mobility of the Ab-loop is significantly related to the ligand’s inhibitory activity (Chi-square test, *p* < 0.05) (Figure 6b). However, 29.2% of the ligand complexes display a closed Ab-loop, similar to the control NMT inhibitors, but have weak activities (Figure 6b), demonstrating that the closing of the Ab-loop is not sufficient for high ligand potency. Interestingly, neither of the ligands with an open Ab-loop (70.8% of the compounds) displays high activity. Thus, the opening of the Ab-loop may lead to weak activity.

Next, we investigated whether the dynamics of the other components of the complex—the cofactor Myr-CoA and the NMT protein—are related to the ligands’ activity. A total of 83.3% of the NMT-ligand complexes display instability in the binding of the cofactor Myr-CoA compared to the control NMT inhibitors (Figure 6c and Figure 7c). The distribution of RMSD of Myr-CoA is significantly related to the inhibitory activity of the ligands (Chi-square test, *p* = 0.001). The increased RMSD of Myr-CoA is manifested by a visible displacement of the cofactor molecule from its binding pocket compared with the control NMT inhibitors (Figure 7h, Movie S4). Considering that there is no direct interaction between Myr-CoA and the ligands, the observed effects could be attributed to conformational changes in the Myr-CoA binding pocket during the MD simulations.

A total of 87.5% of the complexes of ligands identified by the virtual screening have increased RMSD of the whole NMT protein compared to IMP-1088 and DDD85646 complexes (Figure 6d and Figure 7d). The conformational dynamics of the NMT protein are significantly related to the ligand’s inhibitory activity (Chi-square test, *p* < 0.001) (Figure 6d). However, neither RMSD of the Myr-CoA nor the NMT protein alone can be used to unambiguously discern the highly potent ligands from the ones that have weak activity, as 16.7% and 12.5% of the ligands, respectively, display comparable RMSD to the control NMT inhibitors in these components yet display weak activities (Figure 6c,d). In contrast, all ligand-protein complexes with increased RMSD of Myr-CoA or the NMT protein have weak activities.

**Figure 8:**
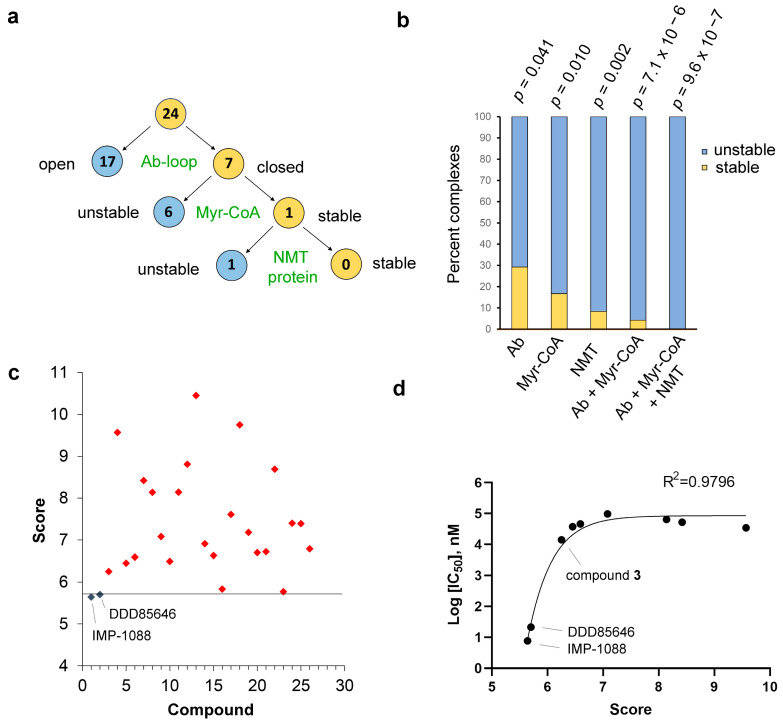
Predicting NMT inhibitor potency based on MD simulations. (**a**) All 24 ligands with weak activity display instability of at least one of the depicted conformational elements compared to the control NMT inhibitors; (**b**) The percent complexes with increased RMSDs of the heavy atoms of the Ab-loop, Myr-CoA, the entire NMT protein, or either of them, compared to DDD85646 and IMP-1088, are shown in blue (unstable); the percent compound complexes with RMSDs of the indicated parameters lower than of the control NMT inhibitors are shown in orange (stable). These distributions are significantly related to the potency of the ligands (Chi-square test, *p*-values are shown on top of the bars); (**c**) The score—the sum of RMSD values of the Ab-loop, Myr-CoA, and the NMT protein for each of the ligand complexes, unambiguously identifies the potent NMT inhibitors DDD85646 and IMP-1088 (blue diamonds) (Chi-square test, *p* < 0.001); (**d**) The score is significantly inversely correlated with the potency of the compounds (logIC_50_) (nonlinear fit, R^2^ = 0.9796). All compounds with IC_50_ < 100 µM (compounds **1** to **9**, Table 1) were used to create the graph. IMP-1088 and DDD85646 are correctly identified as the most potent and second-most potent compounds, respectively. Compound 3 is next in potency and is the best hit from the virtual screening with an IC_50_ = 14 µM (Table 1).

Next, we investigated how the RMSDs of the described conformational elements—Ab-loop, Myr-CoA, and NMT protein—relate. We noticed that the increased RMSD of these elements frequently occurs in complementary ways. For example, 7 of the 24 ligands from the virtual screening (29.2%) have the Ab-loop in its closed conformation yet display weak activity. However, 6 of these 7 ligand complexes have instability in the Myr-CoA binding pocket, and the remaining ligand (compound 16) complex has increased conformational dynamics of the NMT protein (Figure 8a). Analysis of MD simulations of the complex formed by compound 16 shows enhanced dynamics in a specific region. This region is a component of the peptide binding pocket but is separate from the Ab-loop (not shown).

Thus, all 24 ligands with weak activity display instability of at least one of these conformational elements compared to the control NMT inhibitors (Figure 8a,b and Figure S8). This distribution is highly significantly related to the activity of the ligands (Chi-square test, *p* < 0.000001) (Figure 8b).

These results suggest that stabilizing several conformational elements (Ab-loop, Myr-CoA, and NMT protein) simultaneously is required to obtain potent inhibition of NMT. We have also shown that the closed inhibited conformation in the presence of the control NMT inhibitors is almost identical to the structure of NMT in their absence (Figure 4e,f). Hence, it can be concluded that the potent NMT inhibitors stabilize the closed conformation, while the ligands with modest or weak activity destabilize it by opening the Ab-loop or inducing other conformational changes in the protein.

### 2.7. Predicting NMT Inhibitor Potency

We developed a simple scoring function that allows the unambiguous identification of the potent NMT inhibitors from the compounds that display weak or modest activity (Figure 8c). The score represents the sum of the RMSD values of the Ab-loop, the cofactor Myr-CoA, and the NMT protein for each complex (Table S2) and is significantly inversely correlated with ligand potency (Chi-test, *p* < 0.000001) (Figure 8c). Plotting the score values against the IC_50_ of the compounds that have detectable activity (IC_50_ < 100 µM) demonstrated nonlinear regression fit and a significant correlation between the score and inhibitor potency (R^2^ = 0.9796) (Figure 8d). Interestingly, the score correctly identifies IMP-1088 as the most potent ligand and DDD85646 as the second most potent (Figure 8d). The score also correctly identifies the best hit from the virtual screening—compound **3** (Table 1)—as the third most potent ligand (Figure 8d). Plotting the docking scores or change in MM-PBSA free-binding energy (Table 1) instead of the score does not produce curves with statistically significant correlation (R^2^ = 0.0829 and 0.1876 for ChemPLP docking scores and MM-PBSA, respectively), indicating that using the score represents a substantial improvement in predicting the ligand activity compared to the docking scores and the change in free binding energy. Inclusion of RMSD of the ligands did not affect the score’s predictive power (not shown), suggesting that the activity of the NMT inhibitors is primarily correlated with the conformational changes in the protein structure upon ligand binding. These include changes in the Ab-loop’s conformation, the Myr-CoA binding pocket, or the NMT protein as a whole.

### 2.8. A Ligand Entrapment Model of Inhibition

**Figure 9:**
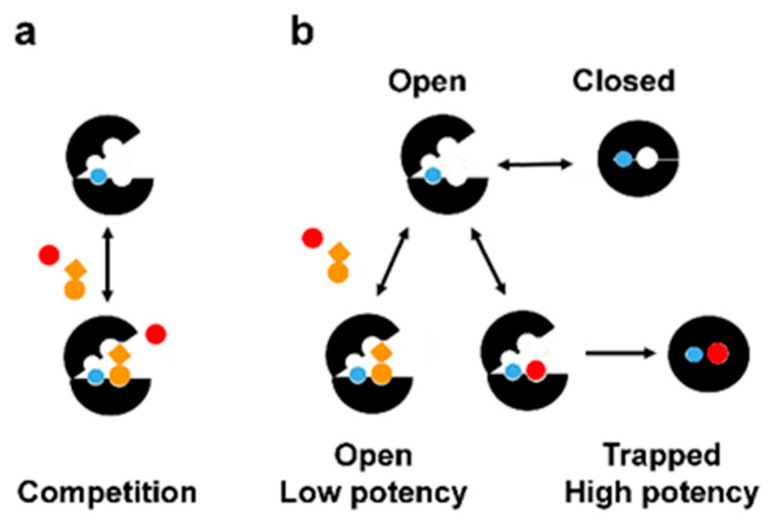
Competitive inhibition vs. ligand entrapment (**a**) A model of competitive inhibition: the binding site is accessible, and the inhibitors compete with the substrate based on the change in free binding energy that arises from their interaction; (**b**) A model of ligand entrapment: the enzyme can adopt open and closed conformations related to different steps of the catalytic process (Figure S9). Inhibitor potency is determined by the ability of the ligand to lock the enzyme in its closed conformation, preventing the accessibility of the substrate to the catalytic center. The enzyme molecule is shown in black, the ligands in red and orange, and the cofactor Myr-CoA in blue. Note that ligand potency varies depending on the inhibition model. The red ligand demonstrates low potency in model (**a**) but high potency in model (**b**), whereas the orange ligand shows low potency in both models but exhibits either higher activity in (**a**) or lower activity in (**b**) compared to the red ligand.

The results presented here suggest that potent inhibition of NMT may occur through several intermittent steps such as (a) the Ab-loop opens, (b) the inhibitor binds in the active site, (c) the Ab-loop closes, and the inhibitor tethers it in a closed conformation due to interaction with its hinge region; (d) the inhibitor “freezes” the enzyme conformation in a closed inhibited state; (e) in this inhibited state the ligand and the cofactor Myr-CoA are trapped inside the protein structure, and their dissociation is severely hindered; (f) the dissociation of the complex requires the dissolution of the closed inhibited conformation, analogously to the processes during dissociation of the products of the enzyme reaction [15]. This mechanism differs substantially from our current understanding of how enzymes or other proteins are inhibited by small molecule ligands. For example, according to the theory of competitive inhibition, the ligands compete for binding to the enzyme’s active site based on the change in free binding energy (Figure 9a) [5,34].

However, the evidence presented here suggests a model of inhibition, according to which the ligand potency is determined by the stabilization of a closed inhibited enzyme conformation (Figure 9b and Figure S9). In this model, the ligands compete based on their ability to keep the enzyme in the closed inhibited state and not simply on their capacity to bind to its active site. A potent inhibition occurs when the ligand becomes entrapped inside the enzyme structure, and hence it becomes severely restrained in its ability to dissociate. Simultaneously, the small molecule’s binding diminishes the enzyme’s dynamic conformational movements to keep it in a closed, inhibited state. In contrast, ligands, which destabilize the closed conformation of the enzyme upon binding, are not trapped and display reduced activity (Figure 9b).

## 3. Discussion

Despite the enormous therapeutic potential, the discovery of drugs with high potency by computational approaches has remained a difficult problem to resolve, and the prediction of inhibitor potency has remained unreliable [4,5]. This was exemplified during the recent COVID-19 pandemic, where the first hits for drug candidates displayed modest activity and lacked the necessary potency to be of clinical significance, eventually leading to a significant delay in the drug development process and the fight against SARS-CoV-2 [6,35,36].

Enzyme inhibition by small molecule ligands is the cornerstone of medicinal chemistry and drug development [37]. Enzymes are known to undergo complex conformational changes during catalysis. In the case of NMT, the opening of the Ab-loop is necessary to accommodate the substrate (peptide) in the active site, and its closing is required for the formation of the catalytically competent form, while its reopening allows the dissociation of the product of the reaction—the myristoylated peptide [15]. Different small molecule ligands may display different affinities for these enzyme conformations. Moreover, depending on the ligand structure, the enzyme may dynamically transition towards its open or closed conformations during the interaction. The outcome—the activity of the ligand—is determined by its ability to stabilize the enzyme in its closed conformation. In this light, this work links the enzyme conformational transitions during catalysis with the capacity of the small molecule ligands to inhibit its function and proposes a novel model for enzyme inhibition (Figure 9b and Figure S9). We refer to this model as “ligand entrapment” or “inhibitor trapping”, emphasizing that the protein molecule functions as a trap that locks the small molecule. However, the ligand also traps the protein in a specific inhibited state by significantly reducing its conformational plasticity.

The results from MD simulations have revealed that the conformational dynamics of the Ab-loop, Myr-CoA, and the NMT protein are significantly related to the activity of the ligands; however, neither of them individually can unambiguously identify the potent NMT inhibitors (Figure 6a–d). Instead, stabilizing the conformation of all these elements together is required to achieve high potency (Figure 8a,b). The effect of stabilization of all conformational elements on the activity of the ligands is highly statistically significant (*p* < 0.000001, Figure 8b), reflecting the finding that all 24 ligands with weak activity display increased instability on at least one conformational element compared to the control NMT inhibitors (Figure 8a). Because of the cooperativity of the conformational elements in mediating ligand potency, the contributions of each appear to be more modest when analyzed individually. For example, the *p*-values for Ab-loop (*p* < 0.05), Myr-CoA (*p* = 0.001), and NMT protein (*p* < 0.001) are several orders higher, compared to the analysis where they are considered together (*p* < 0.000001) (Figure 8b). Hence, predicting the NMT inhibitor activity is possible by creating a scoring function that includes the RMSD of the studied conformational elements together (Figure 8c).

The ligand entrapment mechanism differs from current models of enzyme inhibition, for example, the lock and key mechanism. According to the latter, the ligands or substrate of the reaction (the key) must have a structure that fits precisely in a predetermined binding site of the protein (the lock). According to this model, the binding affinity can be determined by estimating the change in free binding energy that results from the movement of the ligand from the solution to the enzyme’s binding site. This includes estimating the strength of the bonds created between the ligand and the protein and subtracting the effect of the solvation/desolvation of the interacting groups. For the hydrophobic groups of the ligands, the change in free energy represents the effect of desolvation of these groups that may occur in the hydrophobic environment of the protein cavity. Docking score and calculations of free binding energy (for example, by the MM-PBSA method) are modeled on this theory, assuming that the obtained results correlate with the binding affinity. Thus, ligands with high potency are expected to have a more considerable negative change in free binding energy than those with lower potency. However, the current study reveals inconsistencies with this model, leading to several paradoxical observations. First, the docking scores and the MM-PBSA method cannot discern the highly potent NMT inhibitors from those with modest activity or that are even inactive, although they differ by thousands of times in potency (Table 1). Second, this theory cannot explain the synergism between fragments because the change in the free binding energy of two fragments should be additive and not synergistic. Third, the binding of the substrate of the NMT reaction is accompanied by a more substantial change in free binding energy compared to the highly potent NMT inhibitors, raising the question of how these compounds inhibit NMT at all. The model of inhibitor trapping can explain the above inconsistencies. For example, the finding that the substrate peptide participates in more numerous interactions within the binding site of the enzyme than the potent NMT inhibitors, yet it is not inhibitory (Figure S2), could be explained by the fact that the inhibitors are trapped in the structure of NMT, while the substrate peptide is not, and the synergy between complementary binding fragments could occur when one of the fragments stabilizes NMT in its closed conformation (for example, IMP-358 locks the Ab-loop in the closed conformation, Figure 3), trapping the other fragment and making its dissociation more difficult.

The catalytic domain of NMTs is highly evolutionarily conserved, which poses a challenge for developing selective NMT inhibitors. Supporting this observation is that IMP-1088 and DDD85646 inhibit potently both human and protozoan NMTs [16,17], and therefore, using these compounds to treat infectious diseases may cause unintended toxicity by inhibiting the human NMT enzymes. However, even though the binding sites of the protozoan NMTs are highly similar to the human NMTs, a report has described the development of selective inhibitors for NMT from *Leishmania major* [38]. For instance, a derivative compound from the DDD85646 series was found to be 215-fold more potent against LmNMT than HsNMT1 (Ki = 2 nM and 430 nM, resp.) [38]. Through sequence-swapping experiments, it was discovered that these differences were attributed to three amino acid residues positioned at the C-terminus of the proteins. Intriguingly, the side chains of these residues are oriented outward from the binding site and do not engage in interactions with the ligand, raising questions about the mechanism behind the observed selectivity [38]. In light of the current work, the most likely explanation is that these residue side chains affect the protein dynamics and the trapping mechanism in the NMT proteins from different origins. In humans, there are two forms of N-myristoyltransferases: NMT1 and NMT2 [11]. Due to the loss of NMT2 expression in certain tumor types, there is a growing interest in developing selective inhibitors for NMT1 [23]. Although this has not been feasible at the moment because NMT1 and NMT2 have near identical binding sites, the current work provides hope in this area, as it suggests that selective targeting of conserved binding sites may be feasible due to sequence differences in other parts of the protein that affect its dynamic properties. Hence, inhibitor trapping may be of general interest for the design of selective drugs targeting conserved binding sites.

Although it is difficult to estimate how common ligand entrapment is at this time, there are indications that it may occur in diverse enzyme and protein targets. For example, IMP-1088 and DDD85646 were designed by joining fragments that displayed a prominent synergistic effect [16,17]. Similar synergy has been reported in other protein-ligand complexes. For instance, an inhibitor of the matrix metalloproteinase stromelysin (MMP-3) with low nanomolar activity was obtained by joining fragments with micromolar and millimolar binding affinity [39]. Since the synergism is mediated by the trapping of one of the fragments by the other through its effect on the protein conformational dynamics, its presence may indicate the existence of an entrapment mechanism in the studied protein-ligand complex. The synergism between fragments is observed frequently, if not always, when the fragment-based approach has led to the development of highly potent inhibitors, implying that ligand entrapment may be commonplace [39,40,41,42].

Generally, the binding affinity of a ligand, Ka, is determined by the ratio between the binding rate constant k_on_ and the dissociation rate constant k_off_.; Ka = k_on_/k_off_. The trapping of the small molecule is expected to affect its dissociation substantially, resulting in particularly low values of the rate constant K_off_. Consistent with this, very low values of K_off_ have been reported for DDD85646 and IMP-1088 (5.0 × 10^−3^ s^−1^ and 1.9 × 10^−4^ s^−1^, respectively) [16,17], thus providing experimental evidence for ligand entrapment. Low values of the dissociation rate constant k_off_ are a general characteristic of inhibitors with tight-binding kinetics, and indeed, in kinetic assays, the potent NMT inhibitors have been reported to behave as tight-binding inhibitors [16]. In addition, tight-binding inhibition is observed in many other protein complexes, including JAK kinases, COX-1/2, *E. coli* DHFR, *M. tuberculosis* enoyl reductase, and others [43,44,45]. Inhibitor trapping may be the primary mechanism responsible for tight-binding inhibition., as it explains the low dissociation rate. However, it is also possible that the same mechanism may be operational to a lesser extent in many other protein-ligand complexes, where the entrapment is not complete but nevertheless can affect the dissociation rate constant k_off_ and, in turn, the binding affinity of the ligand.

Increased conformational dynamic is expected to diminish the capacity of the protein to trap the ligand inside its structure and facilitate dissociation. Experimental evidence supporting the above hypothesis has been reported. For example, Carroll et al., by using relaxation dispersion, have shown that the dissociation rate and the general affinity constant of E. coli dihydrofolate reductase (DHFR) complexes with different antifolate inhibitors largely depend on conformational movements in the enzyme [46]. Similarly, Seo et al., using single-molecule kinetic analysis, have demonstrated that the dissociation of maltose from its complex with maltose-binding protein is dictated by protein conformational dynamics [47]. These examples are consistent with the model of ligand entrapment.

Other evidence comes from experimentally determined crystallographic strictures that show that different ligands can bind distinct protein conformations. For example, the anti-cancer drug imatinib crystallizes in complex with an inactive conformation of the Abl kinase, known as the ‘DFG—out’ conformation [48], while other ligands, such as dasatinib, are bound to the active ‘DFG—in’ conformation of Abl [49]. Similarly, the drug erlotinib crystallizes in complex with the active conformation of the wt Epidermal Growth factor receptor (EGFR) kinase domain, while lapatinib binds to an inactive form of the receptor, known as the CDK/Src-like inactive conformation [50,51]. Another example comes from the cyclooxygenases COX-1 and COX-2. These enzymes can be targeted by different classes of inhibitors that recognize distinct enzyme conformations [43]. Class I inhibitors are characterized by high potency and very low dissociation rates (tight inhibitors), while class II inhibitors show modest potency and fast dissociation rates [43]. These observations suggest that the different ligands can recognize and trap distinct protein conformations.

The ligand becomes entrapped due to the formation of several specific bonds within the binding site. For example, the interactions of the NMT inhibitors with Ser405 and Phe190 prevent the opening of the Ab-loop to stabilize the closed enzyme conformation (Figure 5). In addition, we have recently found that the salt bridge with the C-terminus of NMT complements this effect by stabilizing the Myr-CoA binding pocket and the overall NMT protein conformation [18]. Hence, during entrapment, the ligand may need to (a) interact with a selected few residues in the active site (for example, S405 and C-terminus in NMT) that stabilize the protein conformation, and (b) its overall structure needs to be compatible with the existence of the closed enzyme conformation to prevent the enzyme from transitioning to its open form. The total number and combined strength of bonds between the ligand and the enzyme may be of secondary significance. The bonds that lead to the entrapment may have a dramatic effect on the affinity of the ligand, exceeding by orders of magnitude the expected increases based on the change in free binding energy. For example, the salt bridge between the NMT inhibitors and the C-terminus of NMT increases the activity of the ligands by over 1300-fold, a much larger change than expected [18]. Similarly, a salt bridge between a ligand and Asp831 in the kinase domain of the Epidermal growth factor receptor (EGFR) is reported to increase the potency 800-fold [52]. In other protein targets, adding a single methyl group to the ligand can boost its activity by hundreds or even thousands of fold, while the change in free binding energy suggests that the change in binding affinity should not exceed 3.5-fold [53]. The mechanism of this so-called ‘magic’ methyl effect is not well understood at the moment, but the inconsistency between the change in free binding energy and activity implies that the effect may depend on the trapping of the ligands facilitated by their extra methyl group.

Although data supporting the model of ligand entrapment is present in the literature, its mechanism has not been illuminated, and no such model has even been proposed. As a result, inhibitor trapping is not currently considered or actively pursued during drug design and development. We believe this is the main reason why inhibitors with high potency cannot be currently designed solely based on available crystallographic structures or computational approaches.

The results reported here pinpoint some faults in the in silico methods currently applied and provide the basis for developing alternative approaches that could accurately predict ligand activities. For instance, the docking scores were found to be unreliable in predicting the activity of the NMT-ligands (Table 1). This could be attributed mainly to the fact that docking does not account for the mechanism of inhibitor trapping. For example, docking is performed using protein structures with rigid backbones, and it is not designed to identify conformational changes in the target protein; for instance, the opening or closing of the molecule upon ligand binding that may indicate whether the ligand is entrapped or not. The fact that docking scores are generally uncorrelated with experiential binding affinity and activity is well known. This has been suggested to be due to assumptions made in the docking algorithms that lead to inaccuracies in estimating the change in free binding energy or the strength of certain bonds [5,8,9]. However, the results presented here suggest that the problem may not be in the docking score calculations per se but may originate from the theory on which docking is based, and which ignores protein dynamics as a factor in the determination of the ligand binding affinity. Conversely, the results presented here suggest that protein dynamics play a critical role in determining the dissociation of the ligand and its overall binding affinity and activity. Therefore, if the docking scores include parameters indicative of the probability of conformational changes in the protein induced by binding (docking) of the ligand, their predictive power could be remarkably improved. It remains to be seen if the development of such “conformational docking” will be feasible.

Here, we have shown the applicability of MD simulations in demonstrating ligand entrapment and predicting NMT inhibitor activity based on the detection of conformational changes in the NMT protein structure due to ligand binding (Figure 8). Apparently, the ligands that stabilize the closed inhibited conformation of the protein are the most potent, and the ones that destabilize this conformation by opening the enzyme structure display reduced potency. The conformational changes induced by the binding of ligands may be confined to specific protein regions that have evolved to possess increased flexibility, such as the Ab-loop of NMT. Thus, determining the conformation of such loop regions in different enzymes may help to predict inhibitor activity, although this may require prior knowledge of the conformational changes of these loops during catalysis. Computer tools that can predict the 3D structures of proteins, such as Alphafold [54], may also be helpful in the prediction of ligand entrapment and inhibitor potency, provided that they can correctly identify the conformational changes in the protein resulting from the binding of the ligand. AlphaFold has made a significant breakthrough in predicting protein 3D structures based on the primary amino acid sequence [54]. Whether this discovery will revolutionize the field of chemical therapeutics depends on our ability to use the available structural information to design ligands that display the desired selectivity and potency. This may become a reality only if the mechanisms driving the inhibition process are well understood.

For a ligand entrapment mechanism, the change in free binding energy ΔG_bind_ is described in Equation (1).

ΔG_bind_ = ΔG_inter_ + ΔG_conf_ (1)

ΔG_inter_ is the change in free energy resulting from the direct interactions between the ligand and the enzyme, and ΔG_conf_ is the change in free energy resulting from conformational changes in the protein during the formation of the closed inhibited state. For competitive inhibitors, ΔG_bind_ is related to the affinity constant K_a_ (binding affinity) and inhibitor potency by Equation (2).

ΔG_bind_ = −RTlnK_a_ (2)

Thus, ΔG_bind_ and, respectively, the binding affinity and potency of the ligands that become entrapped are not determined solely by the direct interactions within the binding site but also depend on the overall protein conformation, particularly on the change in free energy due to the stabilization of the closed enzyme conformation (ΔG_conf_). If |ΔG_conf_| >> |ΔG_inter_|, the binding affinity may depend primarily on the stabilization of the closed enzyme conformation and only secondary on the strength of the direct interactions within the binding site. For ligands that are not entrapped, and the enzyme remains in the open conformation (ΔG_conf_ = 0), the potency may be determined primarily from the direct interaction of the small molecule within the binding site (ΔG_inter_). However, these ligands may display only modest activity, as is the case with the compounds identified in the virtual screening (Table 1).

Because docking is performed with proteins with rigid backbones, docking scores reflect only on ΔG_inter_ and do not account for ΔG_conf_. This could be the main reason the docking scores do not predict the binding affinity correctly, especially when used to compare ligands that are entrapped vs. ligands that are not. Considering that such ligands may differ by thousands of folds in activity, ΔG_conf_ may substantially contribute to the overall binding affinity.

Another way to represent ligand trapping thermodynamically is to consider the reverse reactions of dissociation of the protein-ligand complex, as described in Equation (3).

ΔG_dis_ = ΔG_int_ + ΔG_open_ (3)

ΔGdis is the total energy required for the dissociation of the complex; ΔGint is the energy for disrupting the direct bonds between the ligand and the enzyme; and ΔGopen is the energy necessary for the opening of the enzyme. Because the dissociation of the complex requires energy, the change in ΔGdis is positive, contrary to ΔGbind in Equation (1).

Thus, the energy required for dissociating trapped ligands is the sum of the energy needed to disrupt the direct interactions between the ligand and the protein plus the energy necessary for the transition from the closed to the open enzyme conformation. The energy for the enzyme opening could be supplied by the hydrolysis of the high-energy thioester bond of Myr-CoA (or ATP in kinases, for example) during the catalytic reaction. In contrast, the dissociation of the complexes of the trapped NMT inhibitors may require an equivalent amount of energy to be provided externally, potentially explaining their very low dissociation rate. On the other hand, the dissociation of ligands bound to the open conformation of NMT may not face such a high energy barrier, leading to reduced potency.

## 4. Materials and Methods

Materials and reagents. The NMT inhibitors IMP-1088 (compound **1**) (# 25366, purity ≥ 95%) and DDD85646 (compound **2**) (# 13839, purity ≥ 95%), as well as compound **11** (HTH-01-015, purity ≥ 95%), were obtained from Cayman Chemical (Ann Arbor, Michigan, USA). Compounds **3**, **6**, **7**, **8**, **9**, **12**, **14**, **17**, **18**, **19**, **20**, **21**, **22**, **23**, and **26** were purchased from Asinex Inc. (Amsterdam, the Netherlands). Compounds **4**, **5**, **10**, **13**, **15**, **16**, **24**, and **25** were ordered from Molport (Riga, Latvia). The purity of the screening compounds is estimated to be at least ≥85–90%. The ZINC IDs and the structures of the compounds are included in Table S1. Recombinant full-length human NMT1 protein (# 80R-4067) was purchased from Fitzgerald Industries International. The substrate peptide (Src peptide 2–9) with sequence H-Gly-Ser-Asn-Lys-Ser-Lys-Pro-Lys-NH2 used in the NMT assay corresponds to the N-terminal region of c-Src, with amidation at the C-terminus as indicated, was custom made by GenScript (Rijswijk, The Netherlands). CPM (7-Diethylamino-3- (4-maleimidophenyl)-4-methylcoumarin) and Myristoyl-CoA were purchased from Cayman Chemicals, and the buffer components from Merck (Kenilworth, NJ, USA). Polystyrene Greiner bio 655076, 96-well black, flat bottom, medium-binding, non-sterile plates for performing the NMT assay and the fluorescence reading were purchased from Merck (M4936-40EA).

Visualization of protein-ligand interactions and image preparation. Protein-ligand interactions were visualized in PyMOL 1.6.0.0 (Schrödinger, New York, NY, USA) [55] and YASARA v. 20.4.24 (IMBM, University of Graz, Austria) [56]. The π-π interactions were identified in YASARA. Images were prepared in PyMOL 1.6.0.0 (Schrödinger, New York, NY, USA) [55]. The nomenclature of the secondary structural elements in NMT, such as the names and positions of the α-helices, β-sheets, or connecting loops, was adopted from Dian et al. [15]. The βk’ sheet is disordered in some NMT structures, and it is not described by Dian et al. [15]. We named this β-sheet—βk’, because of its proximity to the βk sheet.

Virtual screening and docking with AutoDock Vina. The virtual screening was performed as previously described [19]. Briefly, 1,114,610 structures with a charge of +1 at reference pH 7.0 were downloaded from the ZINC15 database in pdbqt format in July 2020. The filters used included: reactivity—standard; purchasability—in stock; pH—reference, pH 7.0; charge: +1; MW and LogP—drug-like (compounds with MW 250–500 Da and LogP between −1 and 5). The virtual screening was performed using AutoDock Vina v.1.1.2 [19,57] (The Scripps Research Institute, La Jolla, CA, USA). The ligands were docked into the X-ray structure of HsNMT1 in complex with DDD85646 and Myr-CoA (PDB 3IWE). Prior to docking, DDD85646 was extracted in silico, and the binding site was defined by a grid box with coordinates: center x = −11.077, center y = 21.635, center z = −5.355, size x = 28, size y = 26, and size z = 26. Myr-CoA was kept in the structure and was present during the docking. All partners (NMT protein and Myr-CoA) except ligands were kept rigid during docking. The parameters were set to energy range = 3 and exhaustiveness = 20. The docking was performed on FUJITSU servers with GPUs NVIDIA Tesla V100 at the Faculty of Pharmacy, Medical University of Sofia, and run on the Linux operating system. The docking solutions were visualized using MGL Tools v 1.5.6 [19].

Molecular docking with GOLD. Docking was performed using GOLDsuite version 5.3.0 (CCDC Ltd., Cambridge, UK) [58]. The crystal structure of the ternary HsNMT1: Myr-CoA: DDD85646 complex (PDB 3IWE) was used for docking after the in silico removal of the bound inhibitor. Myr-CoA was present during the docking. The radius of the binding site was set to 6 Å. The protocol was optimized in terms of scoring function, the rigidity/flexibility of amino acid side chains, the presence or absence of water molecules within the binding site, and the number of genetic algorithms (G.A.) runs. Comparable results were obtained irrespective of whether the NMT protein used for docking was rigid or set with flexible side chains or whether the docking was performed in the presence or absence of structural water molecules. Docking with GOLD reproduces with high fidelity the pose, orientation, and interactions in the binding site of the NMT inhibitors compared to the original crystallographic structures. For example, re-docking of IMP-1088 (PDB 5MU6), DDD85646 (PDB 3IWE), and the substrate peptide (PDB 6QRM) produces docking poses that almost perfectly match the poses of the ligands in the crystal structures (RMSD = 0.2095, 0.2564, and 0.1413, respectively) and reproduces all interactions between the ligands and the protein that are present in the X-ray structures. The docking runs in the present study were performed using flexible ligands and the scoring function ChemPLP. The side chains of ten amino acid residues from the binding site, in proximity to the bound ligand (Phe188, Phe311, Tyr296, His298, Y180, Phe190, Asn246, Thr282, Ser405, and Gln496), were set as flexible. Three structural water molecules (HOH2, HOH760, and HOH970) were also present and were set to toggle and spin. The docking was performed in 100 GA runs for each ligand, but only the top 10-scored solutions were saved for analysis.

Molecular dynamics simulations. MD simulations were performed as previously described [19,57]. Before running the MD simulations, all ligands were docked into the structure of the ternary HsNMT1: Myr-CoA: DDD85646 complex (PDB 3IWE) after the extraction of DDD85646 in silico. The best-scored docking solutions were chosen as starting frames for molecular dynamics (MD) simulations. The ligand and Myr-CoA structures were parametrized using the GAFF2.11 force field [59] and AM1-BCC charges [60] using Antechamber from the Amber v.18 package. The NMT protein used for MD simulations contained an N-terminal ACE cap but no C-terminal cap. The addition of a C-terminal cap was not possible because it could have interfered with the interaction with the ligand’s positively charged group and was not necessary because the C-terminus of the native NMT protein contains a negatively charged carboxyl group at this position. The crystal structures of NMT-IMP-1088/ DDD85646 complexes do not contain the full-length NMT protein and have truncated N-terminal parts (the first 114 amino acids). Thus, the addition of the N-terminal ACE cap is necessary to eliminate the positively charged N-terminus at position 115 that is not present in the full-length protein. The complex was solvated in saline (0.9% sodium chloride) in a truncated octahedral box, energy minimized, heated to 310 K at constant volume for 1 ns, density equilibrated at 1 bar for 1 ns, equilibrated keeping constant T and *p* for 1 ns, using the Langevin thermostat [61] and Berendsen barostat [62], and simulated for 1000 ns by AMBER v. 18 (UCSF, San Francisco, CA, USA) [63,64]. During all steps of simulations, i.e., heating, density equilibration, preproduction, and production, the dynamic SHAKE algorithm was used for constraining covalent bonds involving hydrogen with a 2 fs time step [65]. The bonds to hydrogen were not constrained only during the energy minimization step. The systems were simulated with the ff14SB force field [66]⁠ under periodic boundary conditions. Frames were saved every 1 ns to generate 1000 frames for a total of 1 µs duration of MD production simulations. For MD simulations of NMT complexes with complementary synergistic fragments, the crystal structure of *Plasmodium vivax* NMT (PvNMT) in complex with Myr-CoA, IMP-72, and IMP-358 (PDB 5O4V) was used. The MD simulations were performed in the absence of the fragments or in the presence of IMP-72, IMP-358, or both fragments using the above MD protocol. Myr-CoA was present during the MD simulations.

Determination of RMSD of the Ab-loop, Myr-CoA, and NMT protein. The data from the MD simulations were analyzed in VMD (Visual Molecular Dynamics, the University of Illinois at Urbana–Champaign, IL, USA) [67]. The protein used in the crystal structure PDB 3IWE has a deletion of 114 amino acids from the N-terminal part of NMT and is 382 amino acids long, while the full-length HsNMT1 protein is 496 amino acids long. The Ab-loop is the region 180–187 in the full-length HsNMT1, corresponding to residues 66-73 in PDB 3IWE (resid 66 to 73); the ligand (resid 383), Myr-CoA (resid 384), and the NMT protein (resid 1 to 382). RMSD (root-mean-square deviations) of the heavy atoms of the ligands, NMT protein, Ab-loop, and Myr-CoA were determined by the RMSD trajectory tool in VMD.

Scoring function. The score was calculated by adding the RMSD values of the Ab-loop, the Myr-CoA, and the NMT protein for each of the NMT-ligand complexes based on the MD simulations data. The analysis included the two control NMT inhibitors, DDD85646 and IMP-1088, and the 24 compounds identified by virtual screening.

Statistics: Chi-square test and R^2^. In this study, two sets of compounds were compared: the two control NMT inhibitors, DDD85646 and IMP-1088, that are highly potent (IC_50_ in the nanomolar range), and 24 compounds identified by virtual screening that have modest activity (IC_50_ = 14–96 µM) or no activity (IC50 > 100 µM) (Table 1 and Table S1). The Chi-square test *p*-values for the Ab-loop were determined as follows: The Ab-loop in the control inhibitor complexes is in a closed conformation. Thus, complexes with RMSD values of the Ab-loop equal to or lower than those of the control inhibitors (RMSD < 1.5 Å) will have the Ab-loop also in the closed conformation. The complexes with RMSD of the Ab-loop above those of the control inhibitors (RMSD > 1.5 Å) have increased dynamics of the Ab-loop. The null hypothesis is that the conformational mobility of the Ab-loop (its RMSD values) is not related to the activity of the ligands. Then the expectation will be that the distribution of the RMSD of the Ab-loop among the 24 complexes will be random, e.g., 12 will have RMSD below (closed Ab-loop) and 12 above the RMSD values of the control inhibitors (open Ab-loop). In the MD simulations, it was observed that 7 complexes had RMSDs lower than and 17 above the RMSDs of the control inhibitors. The *p*-value in the Chi-square test was calculated in Excel, according to Table 2.

The *p*-value obtained is *p* = 0.041 (*p* < 0.05). The Chi-square test *p*-values for Myr-CoA and NMT protein dynamics were similarly determined. The complex is considered stable if the RMSD of Myr-CoA or NMT protein is equal to or lower than the corresponding values in the control NMT inhibitor complexes, and unstable if it is above these values. A total of 20 of the 24 ligands had an observable increase in RMSD of Myr-CoA, and 21 of the 24 had increased RMSD of the NMT protein compared to the control NMT inhibitors. All 24 ligands had increased RMSD on either the Ab-loop, Myr-CoA, or NMT protein. The significance threshold was set at 0.05.

For the determination of a correlation between the score and the specific IC_50_ values of the compounds, only the compounds that have IC_50_ < 100 µM were used (compounds **1–9**, Table 1) because, for the compounds with IC_50_ > 100 µM, there were no specific IC_50_ values that could be used to create the graphs. The charts and R^2^ (the square of the correlation coefficient) were obtained after plotting the score versus log (IC_50_) for each compound using a nonlinear fit in GraphPad Prism. R^2^ > 0.95 indicates a statistically significant correlation.

MM-PBSA Calculations. Based on MD simulations of each NMT-ligand complex, the change in binding energy was computed using the MM-PBSA (molecular mechanics Poisson–Boltzmann surface area) method and the MMPBSA.py tool [68] from the Amber18 package, as previously described [69,70].

NMT inhibition assay. IC_50_ values were determined using a fluorogenic NMT assay, as previously described [31]. The assay detects Coenzyme A (CoA), which is released as a byproduct of the enzymatic reaction. Briefly, 18.9 nM full-length recombinant HsNMT1 enzyme was incubated in the presence of 3-fold dilutions of the various ligands (4 µM Myristoyl-CoA and 4 µM substrate peptide) and 2.7% DMSO. The reactions were performed in reaction buffer (20 mM potassium phosphate, pH 8.0, 0.5 mM EDTA, 0.1% (*v*/*v*) Triton^®^ X-100) in 44 µL volume in a 96-well black plate. To eliminate the background fluorescence, reactions were also performed in the absence of the NMT1 enzyme for each concentration of the ligands. After 30 min of incubation at 25 °C, 20 µL of CPM in reaction buffer was added to a final concentration of 5.5 µM. The reaction was stopped after 5 min by adding 36 µL of 0.1 M sodium acetate buffer, pH 4.75. Fluorescence was measured at an excitation wavelength of 380 nm and an emission wavelength of 470 nm with a CLARIOstar fluorescent reader (BMG LABTECH GmbH, Ortenberg, Germany). The fluorescent values of samples obtained in the absence of NMT1 were subtracted from the fluorescent signal of samples performed in the presence of NMT1 to eliminate the background signal. The subtracted values were plotted in GraphPad Prism using a log (inhibitor concentration) vs. normalized response nonlinear fit to generate inhibitory curves and determine IC_50_ values. The highest final concentration of the ligands used in the assay was 100 µM, and if this concentration was insufficient to reduce the specific signal by 50% or more, an IC_50_ > 100 µM value was assigned. The experimentally determined IC_50_ cannot be lower than half the enzyme concentration used in the assay, or 9.45 nM for the current assay. Hence, the assay underestimates the potency of IMP-1088 because the reported IC_50_ < 1 nM for IMP-1088 cannot be measured using the enzyme concentrations used in this study. The IC_50_ values of all remaining ligands are within the sensitivity threshold of the assay.

## 5. Conclusions

Our investigation into NMT yielded an intriguing discovery of a novel mechanism of inhibition by small molecules, which we refer to as “ligand entrapment” or “inhibitor trapping”. This mechanism involves the embedding and locking of a small molecule within the enzyme’s structure, resulting in its capture and high potency as an inhibitor. Notably, this process may extend to other enzyme or protein complexes, offering new insights into the broader understanding of how small molecule ligands inhibit their targets.

## Figures and Tables

**Table 1:** Lack of correlation between NMT-ligand potency, docking scores, and MM-PBSA free energy.

	Compound ^1^	IC_50_, µM	Fold ^2^ Potency	% Inhibition ^3^	GOLD ChemPLP	ADV Affinity kcal/mol ^4^	MM-PBSA kcal/mol
1	IMP-1088	0.0076 ± 0.0006	1	100	111.36	−10.8	−56.83
2	DDD85646	0.0213 ± 0.0001	2.8	100	103.59	−11.1	−61.61
3	compound 3	14.0 ± 0.6	1842	98	119.73	−11.6	−59.42
4	compound 4	34.0 ± 2	4474	86	113.09	−11.9	−37.08
5	compound 5	37.5 ± 1.8	4934	82	98.10	−11.4	−57.37
6	compound 6	46.0 ± 4	6053	72	105.16	−11.2	−61.49
7	compound 7	52.0 ± 14	6842	63	94.50	−11.1	−52.96
8	compound 8	64.0 ± 10	8421	60	110.28	−12.0	−36.46
9	compound 9	97.0 ± 20	12,763	51	120.37	−11.4	−48.02
10	compound 10	>100	>13,158	5	113.94	−11.3	−68.82
11	compound 11	>100	>13,158	0	110.07	−12.0	−63.57
12	Substrate peptide	n.a ^5^	n.a ^5^	n.a ^5^	142.83	−20.3	−78.71

^1^ Compounds **1** and **2** are IMP-1088 and DDD85646, ^2^ The ratio of IC_50_ of each compound to IC_50_ of IMP-1088, ^3^ Percent inhibition at concentration of the compounds 100 µM, ^4^ ADV—AutoDock Vina, ^5^ Not applicable.

**Table 2:** Calculations of Chi-square test *p*-values for the Ab-loop.

	Ab-Loop Closed	Ab-Loop Open
Observed	7	17
Expected	12	12
Chi-square test	*p* = 0.041	

## Data Availability

Not applicable.

## Supplementary Materials

The following supporting information can be downloaded at: https://www.mdpi.com/article/10.3390/ijms241411610/s1.
