# Supplementary material for: Inhibitor Trapping in N-Myristoyltransferases as a Mechanism for Drug Potency
Source: Int J Mol Sci. 2023 Jul 18;24(14):11610. doi: 10.3390/ijms241411610 (PMC10380619; doi:10.3390/ijms241411610)
Supplement: Supplementary file 1 [file ijms-24-11610-s001.zip › ijms-2499732-supplementary.pdf]

Supplementary Materials:

Inhibitor Trapping in N-Myristoyltransferases as a Mechanism for Drug Potency

Danislav S. Spassov *, Mariyana Atanasova and Irini Doytchinova

Department of Chemistry, Faculty of Pharmacy, Medical University of Sofia, 1000 Sofia, Bulgaria; matanasova@pharmfac.mu-sofia.bg (M.A.); idoytchinova@pharmfac.mu-sofia.bg (I.D.)
* Correspondence: dspassov@pharmfac.mu-sofia.bg

Table S1. Structures and potency of NMT ligands used in this study. The order of the compounds in the table is the same as used in Table 1, Fig. 7a-d, and Fig. 8c.

Compound	Name	IC_{50}, μM	Structure	GOLD ChemPLP	ADV affinity kcal/mol
1	IMP-1088	0.0076	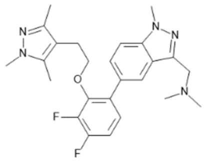	111.36	-10.8
2	DDD85646	0.0213	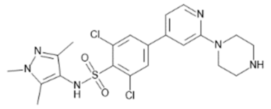	103.59	-11.1
3	ZINC19710136	14.0	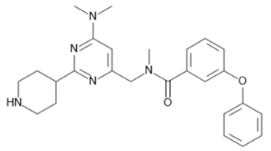	119.73	-11.6
4	ZINC61997750	34.0	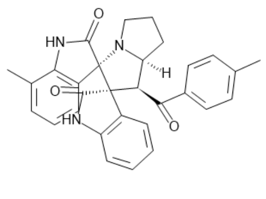	113.09	-11.9
5	ZINC67688793	37.5	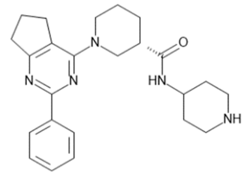	98.10	-11.4

6	ZINC19708540	46.0	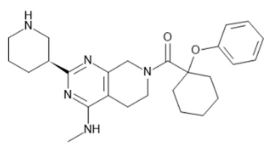	105.16	-11.2
7	ZINC19566088	52.0	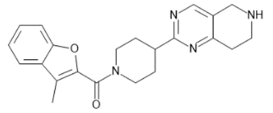	94.50	-11.1
8	ZINC19692195	64.0	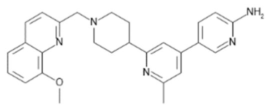	110.28	-12.0
9	ZINC19710084	97.0	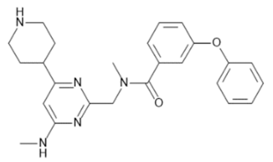	120.37	-11.4
10	ZINC21711800	>100	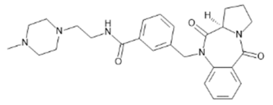	113.94	-11.3
11	ZINC214463354	>100	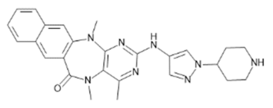	110.07	-12.0
12	ZINC19710116	>100	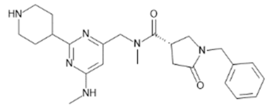	117.55	-11.3
13	ZINC35458799	>100	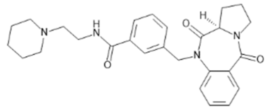	112.31	-11.3
14	ZINC19590209	>100	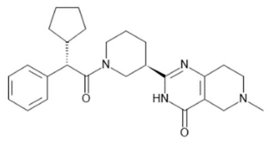	110.20	-11.9
15	ZINC299757951	>100	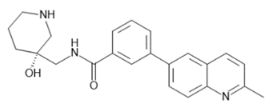	106.95	-11.5

16	ZINC35459261	>100	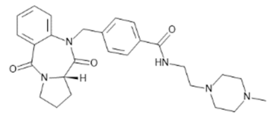	105.64	-11.7
17	ZINC19708784	>100	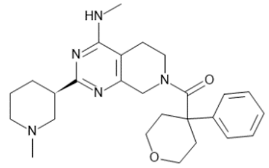	101.21	-11.6
18	ZINC72370170	>100	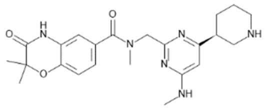	102.19	-11.5
19	ZINC19710924	>100	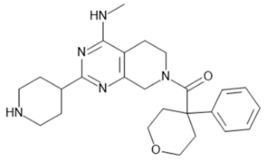	102.12	-11.4
20	ZINC19691948	>100	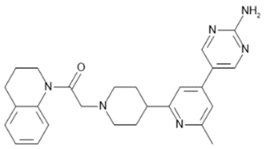	120.17	-12.0
21	ZINC19376075	>100	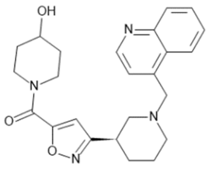	107.51	-12.2
22	ZINC72353749	>100	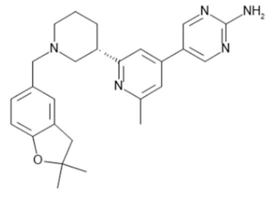	106.19	-11.7
23	ZINC257297002	>100	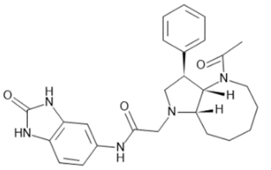	105.31	-12.0

24	ZINC19228549	>100	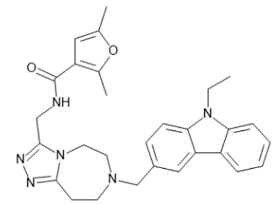	117.52	-11.9
25	ZINC8992179	>100	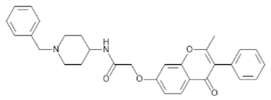	108.0	-11.6
26	ZINC257248718	>100	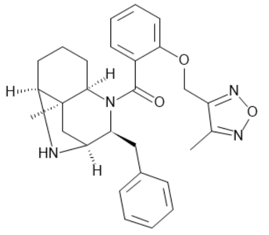	107.29	-11.9

Table S2. Average RMSD values of the heavy atoms of Ab-loop, Myr-CoA, and NMT protein in Angstroms (Å) during MD simulation of complexes of the indicated compounds with HsNMT1. The score represents the sum of the RMSD values for the Ab-loop, Myr-CoA and NMT protein for each complex.

	Compound	RMSD Ab-loop, Å	RMSD Myr-CoA, Å	RMSD NMT protein, Å	Score Sum, Å
1	IMP-1088	1.38	2.28	1.98	5.64
2	DDD85646	1.39	2.12	2.19	5.70
3	Compound **3**	1.58	2.45	2.22	6.25
4	Compound **4**	2.70	4.07	2.80	9.57
5	Compound **5**	1.55	2.69	2.21	6.45
6	Compound **6**	1.34	2.47	2.78	6.59
7	Compound **7**	1.94	4.06	2.42	8.42
8	Compound **8**	2.93	3.00	2.21	8.14
9	Compound **9**	1.36	3.24	2.48	7.08
10	Compound **10**	1.37	2.52	2.60	6.49
11	Compound **11**	1.55	3.85	2.74	8.14
12	Compound **12**	1.32	4.75	2.74	8.81
13	Compound **13**	4.34	3.50	2.61	10.45
14	Compound **14**	1.19	3.36	2.36	6.91
15	Compound **15**	1.54	2.97	2.12	6.63
16	Compound **16***	1.19	2.14	2.50	5.83
17	Compound **17**	1.64	3.77	2.20	7.61
18	Compound **18**	3.32	3.73	2.70	9.75
19	Compound **19**	1.12	3.71	2.35	7.18
20	Compound **20**	1.66	2.89	2.15	6.70
21	Compound **21**	1.51	3.31	1.90	6.72
22	Compound **22**	2.53	3.61	2.55	8.69
23	Compound **23***	1.51	1.87	2.39	5.77
24	Compound **24**	3.48	1.28	2.64	7.40
25	Compound **25**	2.15	2.72	2.52	7.39
26	Compound **26**	2.46	2.11	2.22	6.79

*the NMT complexes with these compounds display increased dynamics of a distinct region in the peptide binding pocket.

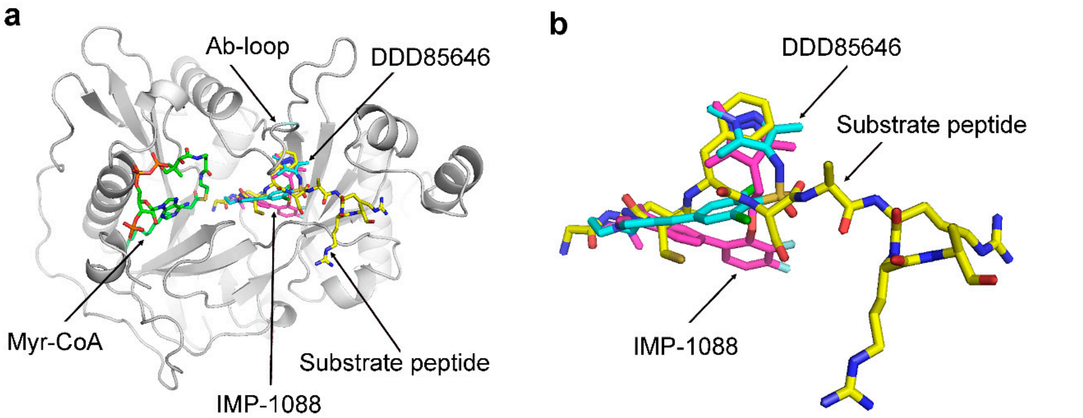

Figure S1. The substrate peptide and the NMT inhibitors occupy the same binding site. Structural superimposition of crystal structures of HsNMT1 in complex with DDD85646 (PDB 3IWE), with IMP-1088 (PDB 5MU6), or with the substrate peptide GNCFSKRRA (PDB 6QRM). The carbon atoms of the substrate peptide are shown in yellow, DDD85646 in cyan, IMP-1088 in magenta, and Myr-CoA in green; (**a)** A view of the whole molecule shows that IMP-1088 and DDD85646 occupy the peptide binding pocket of NMT; (**b**) A zoomed view depicting only the superimposed NMT inhibitors and the substrate peptide.

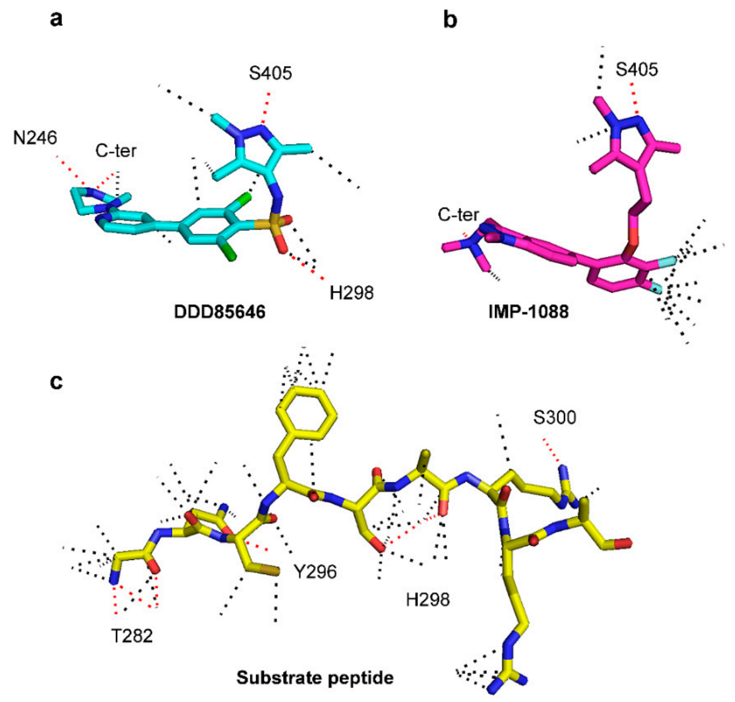

d

Interactions	DDD85646	IMP-1088	Substrate peptide
polar	4	2	7
non-polar	9	15	44
total	13	17	51

Figure S2. The substrate peptide participates in more numerous interactions with NMT than the potent NMT inhibitors. Polar interactions are indicated by red dots and non-polar contacts by black dots. The residues from NMT involved in polar interactions are shown, but those that participate in non-polar contacts are omitted for simplicity. Results were obtained in PyMOL; (**a**) Interactions between HsNMT1 and DDD85646, based on crystal structure PDB 3IWE;(**b**) Interactions between HsNMT1 and IMP-1088, based on crystal structure PDB 5MU6; (**c**) Interactions between HsNMT1 and the substrate peptide GNCFSKRRA, based on crystal structure PDB 6QRM; (**d**) The number of interactions between the indicated ligands and NMT protein, based on figure a-c. Consistent with the higher number of interactions visible in the X-ray structures, the substrate peptide receives a higher docking score in GOLD, a more negative AutoDock Vina affinity score, and a more negative change in free binding energy by MM-PBSA than the potent NMT inhibitors (Table 1).

a

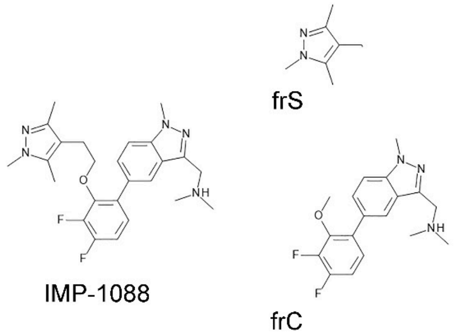

b

Ligand	ChemPLP	GOLDscore	RMSD, Å
frC	85.29	62.50	0.2634
frS	31.31	26.96	0.3023
frC + frS (sum)	116.60	89.46	-
IMP-1088	116.62	88.23	0.2095

Figure S3. Docking scores are additive, and docking algorithms do not calculate synergistic effects. **(a)** The structure of IMP-1088 was split *in silico* into two fragments - frC, which interacts with the C-terminus of NMT, and FrS, which forms a hydrogen bond with Ser405. These fragments and the structure of IMP-1088 were re-docked into the crystal structure of the ternary HsNMT1:Myr-CoA:IMP-1088 complex (PDB 5MU6) after the initial extraction of IMP-1088 from the binding site by using GOLD software; (**b**) The ChemPLP and GOLDscores of IMP-1088 are approximately the sum of the scores of FrC and FrS. The docking poses of the fragments and IMP-1088 were almost identical to the position of the inhibitor in the original crystallographic structure, as indicated by the low RMSD values shown in the last column.

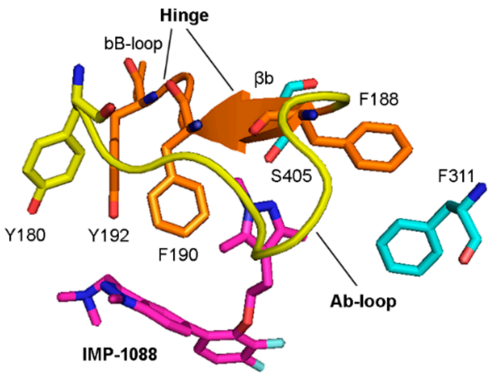

Figure S4. Defining the hinge region of the Ab-loop. The Ab-loop (yellow) and the hinge (orange) are depicted in the crystal structure of the complex between IMP-1088 (magenta) and HsNMT1 (PDB 5MU6). The hinge region contains the sequence $F^{188}RF^{190}DY^{192,}$ with the indicated aromatic residues occurring on every other position. During the opening of the Ab-loop, F188, F190, and Y192 undergo substantial conformational movements (Figure 5b, Figure S5b). The hinge region is immediately connected to the Ab-loop and consists of the small βb-sheet and bB-loop. Other residues undergoing conformational changes during the opening of the Ab-loop but that are not formally a part of the hinge region are also indicated, including Y180 from the Ab-loop. S405 and F311, shown in cyan, are located in different regions in the primary sequence but are near the hinge region in the three-dimensional structure of the NMT protein. The position of the Ab-loop and hinge region in the context of the whole structure of NMT is depicted in Figure 1.

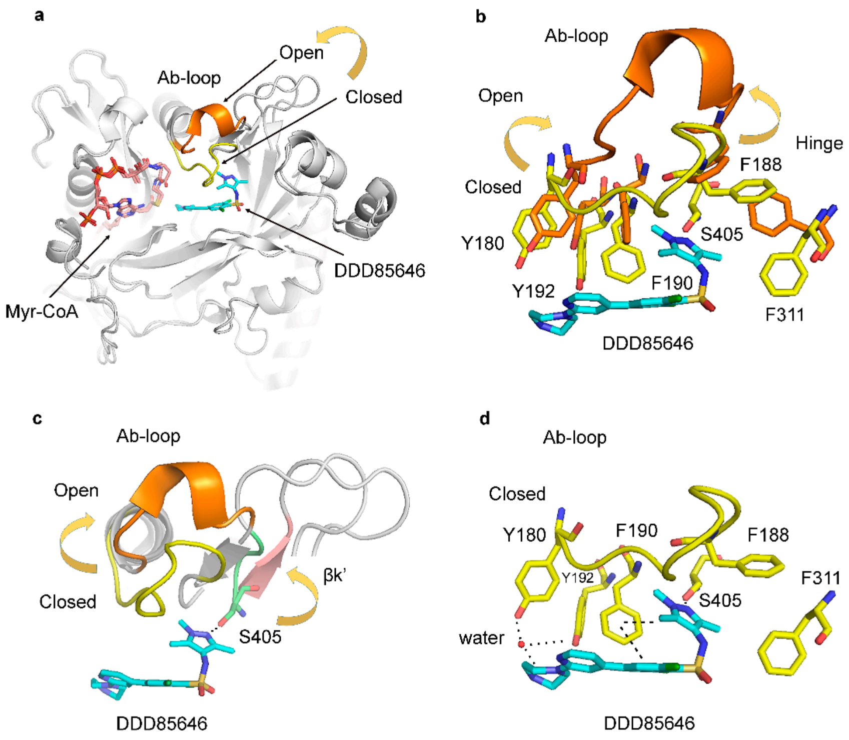

Figure S5. The NMT inhibitor DDD85646 interacts with the hinge region of the Ab-loop. **(a-c)** Structural superimposition of binary ScNMT: Myr-CoA complex (PDB 1IIC) with Ab-loop in the open conformation (orange) and ternary HsNMT1: Myr-CoA: DDD85646 complex (PDB 3IWE) with Ab-loop in close conformation (yellow); (**a)** Whole NMT molecule**; (b)** A zoomed view of the open (orange) and closed (yellow) Ab-loop conformations; (**c)** Conformational movement of S405 due to the opening of the Ab-loop. The formation of a hydrogen bond between the inhibitor and S405 (black dots) is expected to hinder this movement and stabilize the closed Ab-loop conformation; (**d)** Interactions between DDD85646 and residues at the hinge region of the closed Ab-loop. The hydrogen bond between the inhibitor and S405, the dual stacking interactions with F190, and the water bridge with Y180 and Y192 may help keep the Ab-loop in a closed conformation.

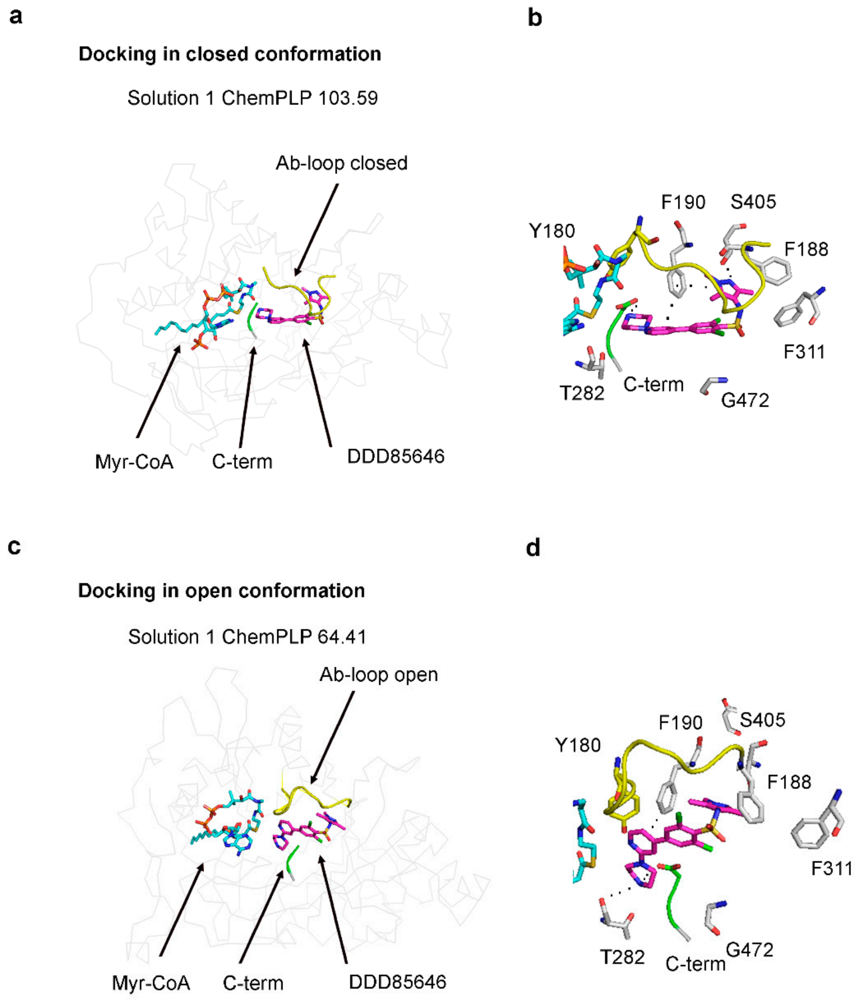

Figure S6. The best scoring solutions for DDD85646 after docking in the closed or open conformation of HsNMT1 using GOLD. Images on the left show a view of the whole molecule, and images on the right a zoomed view of the binding site**.** The Ab-loop is shown in yellow, DDD85646 in magenta, Myr-CoA in cyan, and C-terminus in green; **(a-b)** Docking of DDD85646 in the closed NMT conformation. The docking reproduces the binding pose of DDD85646 in the crystal structure PDB 3IWE (Figure S5). The salt bridge with the C-terminus of the protein, hydrogen bond with Ser405, and double stacking interactions with Phe190 are indicated; (**c-d**) Docking of DDD85646 in the open conformation of NMT. The salt bridge with the C-terminus of NMT is preserved. However, the hydrogen bond with Ser405 and the stacking interaction between the pyrazole ring of the inhibitor and Phe190 is lost. An additional hydrogen bond with Thr282 can be identified. Note that the docking score (ChemPLP) of DDD85646 in the open NMT conformation is substantially lower than in the closed conformation.

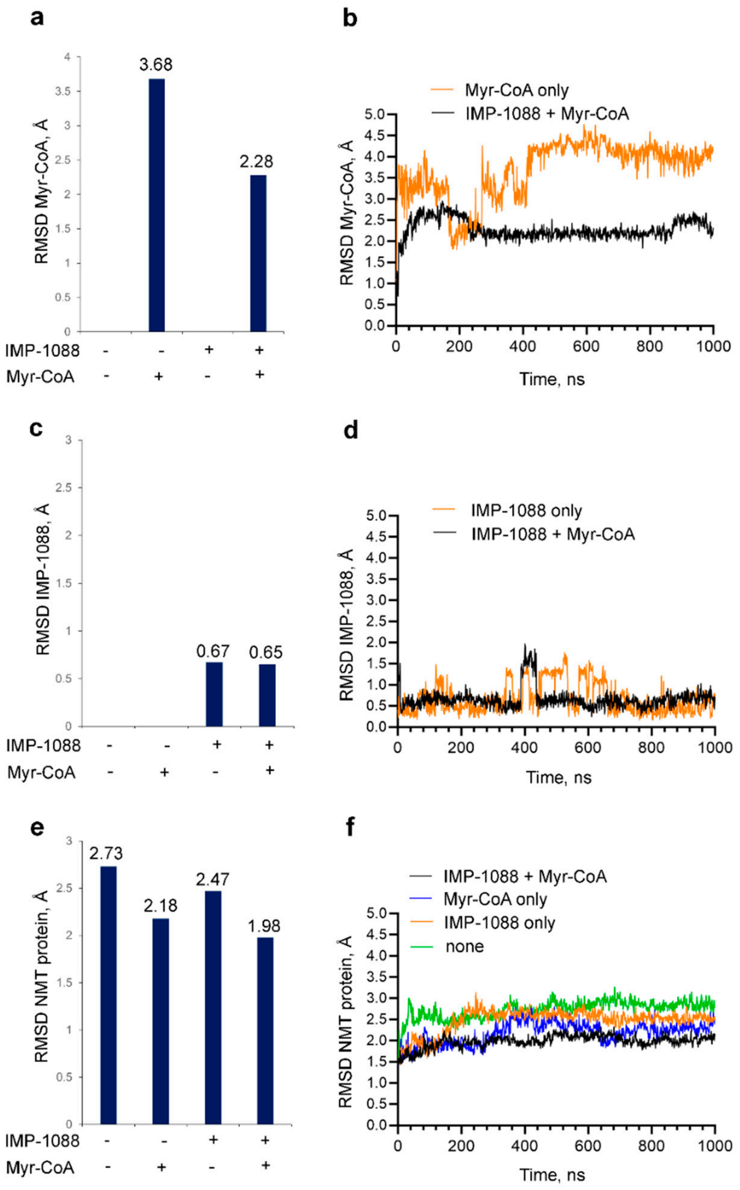

Figure S7. A crosstalk between NMT inhibitors, Myr-CoA, and NMT protein. Results are based on MD simulations of the HsNMT1 in the absence of ligands (none), or in complex with Myr-CoA, IMP-1088, or both. Average RMSD of the heavy atoms of Myr-CoA, NMT protein, and IMP-1088 are shown in the bar graphs on the left, and RMSD values in the time course of MD simulations are depicted on the charts on the right**; (a-b)** The presence of IMP-1088 leads to the stabilization of the complex between NMT and Myr-CoA**; (c-d)** Effect of Myr-CoA on the stability of the complex between NMT and IMP-1088; (**e-f)** Myr-CoA and IMP-1088 reduce the conformational mobility of the NMT protein. The lowest RMSD for the NMT protein is obtained in the ternary NMT: Myr-CoA: IMP1088 complex**.**

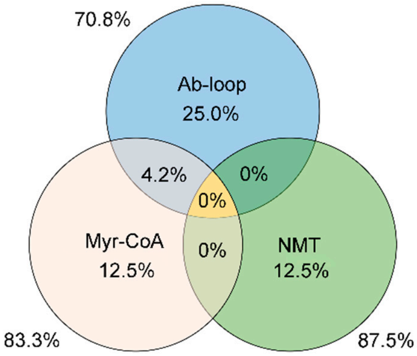

Figure S8. A Venn diagram depicting the distribution of the HsNMT1 complexes with the ligands identified by virtual screening based on RMSD of the Ab-loop, Myr-CoA, and NMT protein. The numbers indicated inside the circles represent the percentage of compounds with RMSD values < RMSD of the control NMT inhibitors DDD85646 and IMP-1088 and the ones outside the circles with RMSD values > RMSD of the control NMT inhibitors. The latter is indicative of increased conformational mobility. The intersection between the three circles, shown in orange, gives an area where the protein conformation is most stable and where the inhibitors are predicted to have the highest potency. Only IMP-1088 and DDD85646 are positioned in this area; all compounds from the virtual screening are outside, as indicated by 0%.

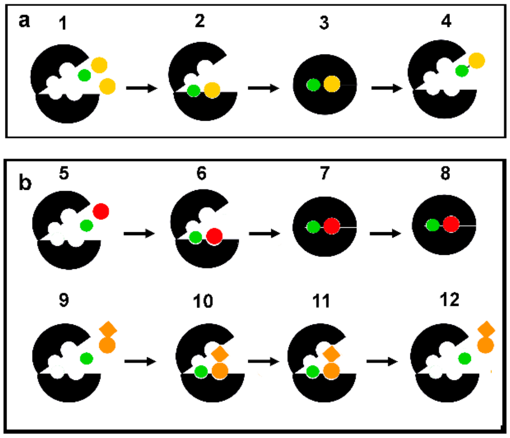

Figure S9. A model of NMT catalysis and inhibition. NMT is shown in black, its cofactor Myr-CoA in green, the substrate peptide in yellow, and the inhibitors in red and orange; (**a)** A model of the NMT catalytic process. NMT can adopt open and closed conformation. When the enzyme is in its open conformation, the active site is accessible, and the cofactor – Myr-CoA and the substrate peptide can bind (1,2). The closed conformation (3) is the catalytically active conformation, and its formation is required for the myristoylation reaction. Dissociation of the products of the reaction requires the reopening of the enzyme (4), which may be facilitated by hydrolysis of the high-energy thioester bond present in Myr-CoA; (**b)** A ligand trap model for inhibition of NMT. The ligand on the top row (5-8) stabilizes the closed NMT conformation and blocks the reopening of the enzyme and dissociation of the complex (8), leading to very potent inhibition. The ligand on the bottom row (9-12) is not compatible with the closed conformation, and by preventing its formation (11), it makes the dissociation of the complex favourable (12), leading to reduced potency.

Supplementary materials movie legends:

Each movie is based on all 1000 frames of MD simulations of complexes of HsNMT1 proteins with the indicated ligands. Each frame corresponds to 1 ns, and the total duration of the MD simulations is 1 μs. The NMT protein is represented by a cyan cartoon with defined secondary structural elements; the ligands are shown in red, the cofactor Myr-CoA in magenta; the Ab-loop is in yellow; Asp184, which is in the middle of the Ab-loop, is shown in orange and Arg255 is in blue.

Movie S1: MD simulations of HsNMT1: IMP1088 complex. Note that the Ab-loop adopts the closed conformation during the entire duration of the MD simulations. The cofactor Myr-CoA is restrained to its binding pocket, and the NMT protein appears more static than the other NMT-ligand complexes.

Movie S2: MD simulations of HsNMT1: compound **18** complex. The Ab-loop opens during the MD simulations and later is stabilized in its open conformation by the formation of a salt bridge between Asp184 and Arg255. The NMT protein is more dynamic than in its complexes with the potent NMT inhibitors.

Movie S3: MD simulations of HsNMT1: compound **24** complex. The ligand completely dissociates from the complex. The Ab-loop opens, and the salt bridge between Asp184 and Arg255 is formed.

Movie S4: MD simulations of HsNMT1: compound **12** complex. The Ab-loop remains in the closed conformation, but the cofactor Myr-CoA is partially displaced from its binding site.
